# The impacts of agroforestry on agricultural productivity, ecosystem services, and human well‐being in low‐and middle‐income countries: An evidence and gap map

**DOI:** 10.1002/cl2.1066

**Published:** 2019-12-23

**Authors:** Daniel C. Miller, Pablo J. Ordoñez, Sarah E. Brown, Samantha Forrest, Noé J. Nava, Karl Hughes, Kathy Baylis

**Affiliations:** ^1^ Department of Natural Resources and Environmental Sciences University of Illinois at Urbana‐Champaign Urbana Illinois 61801; ^2^ Department of Agricultural and Consumer Economics University of Illinois at Urbana‐Champaign Urbana Illinois 61801; ^3^ World Agroforestry Center (ICRAF) Nairobi Kenya

**Abbreviations**
BACI: before‐after‐control‐impact
DID: difference‐in‐difference
EGM: evidence and gap map
FAO: Food Agriculture Organization
FMNR: farmer‐managed natural regeneration
ICRAF: World Agroforestry Center
IPCC: Intergovernmental Panel on Climate Change
IV: instrumental variable
L&MICs: low‐ and middle‐income countries
HIC: high‐income country
NGO: nongovernmental organizations
OLS: ordinary least squares
PES: payment for ecosystem services
PICO: population, interventions, comparison type, and outcomes
PRISMA: preferred reporting items for systematic reviews and meta‐analyses
PSM: propensity score matching
RCT: randomized controlled trial
RDD: regression discontinuity design
SDG: sustainable development goal
SM: systematic map
SR: systematic review

## 1 PLAIN LANGUAGE SUMMARY

### 1.1 Mapping the evidence of agroforestry's impacts on agricultural productivity, ecosystem services, and human well‐being in low‐ and middle‐income countries (L&MICs)

Agroforestry practices have been widely studied across L&MICs, but rigorous evidence on the effects of interventions designed to promote and support agroforestry on farmers’ land remains limited.

### 1.2 What is this evidence and gap map (EGM) about?

Agroforestry, defined as the integration of trees and woody shrubs in crop and livestock production systems, is widely promoted as an effective means to address conservation and development objectives across the world.

Governments, donors, and nongovernmental organizations (NGOs) have invested in a range of programs to spur agroforestry adoption, including farmer capacity development, tree germplasm provision, market development, and community advocacy. However, systematic understanding of the impacts of these programs and agroforestry practices more generally remains lacking.

To advance such understanding, this EGM collates existing evidence on the impacts of agroforestry on agricultural productivity, ecosystem services, and human well‐being in L&MICs. The EGM includes studies that compared farmers and farms where agroforestry was practised to those without agroforestry, to assess at least one dimension of agricultural productivity, ecosystem services, and human well‐being.

What is the aim of this EGM?

This Campbell EGM presents the existing evidence for the impacts of agroforestry practices and interventions on agricultural productivity, ecosystem services, and human well‐being compared to conventional agricultural or forestry practices. Unlike a systematic review, an EGM identifies what evidence exists, rather than summarizing effect size estimates.

### 1.3 What studies are included?

This EGM includes studies that evaluate the effects of agroforestry practices and interventions on agricultural productivity, ecosystem services, and human well‐being.

A total of 20,271 studies were identified. Only 396 of these met the inclusion criteria to be retained for the EGM. Of these studies, 344 examined the effects of agroforestry practices only, 40 examined the effects of agroforestry interventions, and 12 were systematic reviews (SRs). The studies spanned the period from 2000 to mid‐2017, with India, Indonesia, China, and Ethiopia the most studied countries.

Most of the studies were observational. Only eight studies used rigorous quasi‐experimental methods to evaluate the impacts of agroforestry interventions. None of the included studies used experimental designs (random assignment).

### 1.4 What are the main findings of this EGM?

The eight impact evaluations came from different country contexts, with only Kenya having more than one study. The most studied interventions were incentive provision to motivate farmers to plant and maintain trees on their land, and farmer capacity development.

Human well‐being, particularly income and household expenditure, was the most studied outcome category for impact evaluations, followed by impacts on agricultural productivity, with minimal evidence for ecosystem services outcomes.

Practices relating to the integration of crops and trees (agrisilviculture) comprised more than three quarters of the 344 studies on practices. In contrast to the intervention studies, ecosystem services was the most well‐studied practice outcome category, followed by agricultural productivity, with minimal evidence for human well‐being outcomes.

Of the 12 included SRs focused on agroforestry practices, 11 were rated as high risk of bias, and only one was rated as medium risk of bias. Trees integrated with plantation crops was the most common agroforestry practice discussed in the reviews while ecosystem services was the most studied outcome.

No SR examined the effects of agroforestry on human well‐being.

### 1.5 What do the findings of the EGM mean?

Our study reveals that rigorous evidence on the effects of agroforestry interventions on farmers’ land remains extremely limited. This finding is especially notable given the large volume of literature documenting the uptake of specific agroforestry practices and widespread promotion of agroforestry as a strategy to advance the 2030 UN Sustainable Development Goals (SDGs).

The most urgent need in this field is to address the gap in primary evidence on the impacts of agroforestry interventions and on the impacts of agroforestry on social and economic outcomes. SR of the available studies on intervention impacts would be useful to establish a baseline and provide insights to inform future research, policy, and programming relating to agroforestry.

### 1.6 How up‐to‐date is this review?

The review authors searched for studies from 2000 to mid‐2017.

## 2 EXECUTIVE SUMMARY

### 2.1 Background

Agroforestry—the integration of trees with other agricultural practices on the same piece of land—is widespread across L&MICs. High‐level policy documents in many L&MICs explicitly promote agroforestry and donors have invested billions of dollars in agroforestry interventions. Given its potential to boost food security while delivering other social and environmental objectives, agroforestry is seen as a key means to advance the 2030 UN SDGs.

Despite a large body of agroforestry experience in L&MICs, systematic understanding of the social‐ecological impacts of agroforestry remains lacking. This report summarizes the findings of an EGM to address this knowledge need. The EGM identifies, maps, and describes available evidence on the effect of agroforestry on agricultural productivity, ecosystem services, and human well‐being in L&MICs, in addition to the evidence assessing the relationship between agroforestry practices and such outcomes.

### 2.2 Search methods and selection criteria

We systematically identified and mapped evidence on the effects of agroforestry in L&MICs according to a framework that included four broad practice types and six intervention types together with the three outcome categories of agricultural productivity, ecosystem services, and human well‐being. We used a population, intervention, comparator, and outcome (PICO) framework as a basis for inclusion of studies in the EGM. The study population was farms and farming households in L&MICs. “Interventions” in this context included both interventions promoting agroforestry and studies of agroforestry practices applied by farmers in the absence of an external intervention. An alternative intervention or “business as usual” were both eligible comparators. Studies had to measure at least one outcome in the broad categories of agricultural productivity, ecosystem services, or human well‐being.

The decision to include studies of practices in the absence of interventions was motivated by the key role and prevalence of such studies relative to intervention research. The study design inclusion criteria reflect this choice. We included three types of studies: (a) quantitative impact evaluations, (b) SRs, and (c) observational studies. We excluded field trials that did not take place on farmer‐managed land as our focus was on agroforestry effectiveness in “real world” settings. Results for studies assessing interventions and practices were analyzed and presented separately.

To identify potential studies for inclusion we followed the search strategy from a published research protocol (Miller, Ordonez, Baylis, Hughes, & Rana, 2017). In October 2017, we searched six databases and 19 organization websites to identify potentially relevant studies published in English from 2000 to June 30, 2017. Search results were uploaded to EPPI Reviewer v4. A team of 14 reviewers were involved in study screening and data extraction. We first screened articles at title and abstract level and then screened the remaining studies at the full text level. A subset of studies (~10%) was double screened at title/abstract level by the lead reviewers. *κ* tests were used to ensure agreement among reviewers. At the full text level, results were spot‐checked and all data extraction checked for accuracy by lead researchers.

For each included study, we extracted the following data: bibliometric information, study description, information about the agroforestry intervention/practice, the study design and type, information on the outcome and indicator variables, and descriptions of any mechanism describing pathways between intervention and outcome. Only the included SRs were subject to critical appraisal; however, we recorded information about the study design type (e.g., experimental, quasiexperimental, before‐after‐control‐impact, correlational) as an indicator of potential bias. We conducted quantitative analyses using R to create visual representations of our findings in the form of heatmaps and graphs.

### 2.3 Data collection and analysis

Our search returned 20,271 studies, of which 3,080 were removed as duplicates, leaving 16,535 studies that were screened on title and abstract. After title and abstract screening, there remained 1,557 studies which were screened at full text. We identified 12 SRs and 384 primary studies that met our inclusion criteria. Of the primary studies, 40 studies examined the impacts of specific agroforestry interventions, of which only eight used quantitative impact evaluation methods. The other 32 intervention studies measured the outcomes of an agroforestry intervention against a comparator, but they did not use experimental or quasiexperimental methods to account for nonrandom assignment to treatment and control groups. The other 344 primary studies examined the outcomes of agroforestry practices (without a specific intervention associated with the practice) against a nonagroforestry comparator.

The eight impact evaluations came from different country contexts, with only Kenya yielding more than one study. Together, they examined four of the six intervention types in the EGM. The most studied interventions were incentive provision to motivate farmers to plant and maintain trees on their land (*n* = 4) and farmer capacity development (*n* = 4). Two studies included a component of enhancing access to tree germplasm, and one study included a community‐level campaign and advocacy component. Several studies examined interventions with multiple components (e.g., incentive provision and farmer capacity development), so total intervention counts sum to more than the eight studies. We found no impact evaluations of two intervention types: market linkage facilitation and institutional and policy change. Human well‐being, particularly income and household expenditure, was the most studied outcome category for impact evaluations, with five studies examining these aspects. Four studies assessed impacts on agricultural productivity, while only two focused on ecosystem services outcomes.

Of the 12 included SRs focused on agroforestry practices, 11 were rated as high risk of bias, and only one was rated as medium risk of bias. Trees integrated with plantation crops was the most common agroforestry practice discussed in the reviews while ecosystem services was the most studied outcome. No SR examined the effects of agroforestry on human well‐being.

The 344 studies of agroforestry practices were relatively evenly spread across the major tropical and subtropical world regions, though some countries were relatively well studied (e.g., India, Indonesia, China, and Ethiopia, which collectively represent 45% of the total studies). There were hardly any studies from L&MICs in Europe and Central Asia and Middle East and North Africa regions. Practices relating to the integration of crops and trees—agrisilviculture—comprised more than three quarters (78%; *n* = 271) of the 344 studies on practices. In contrast to the intervention studies, ecosystem services was the most well‐studied practice outcome category. The vast majority of included primary studies (96%) were correlation only studies that did not use an experimental, quasiexperimental, or other before‐after‐control‐impact study design. All included studies did have a control group using a nonagroforestry practice (e.g., relating to agriculture or forestry) to compare impacts.

The research on agroforestry practices has grown steadily, from <10 relevant studies in 2000 to nearly 50 in 2016. However, the volume of evidence on agroforestry interventions remained spotty and flat during the study period. Given our inclusion criteria, we did not include field trials, but our search revealed approximately 1,700 potentially relevant studies reporting on results of field trials or “efficacy studies.” A central finding of this EGM is that the evidence base on the effects of interventions promoting agroforestry on farmers’ land remains very limited. This result contrasts to the availability of hundreds of observational and experimental studies on the effect of agroforestry practices. Part of the reason for this finding is the complexity of many agroforestry systems and the relatively long time horizons required for interventions to generate results. For example, it may take many years beyond the scope of an intervention for trees to mature so that they yield useful products such as fruit, fodder, or timber, challenging efforts to monitor and evaluate intervention impacts beyond adoption of promoted practices.

### 2.4 Results and authors’ conclusions

Our study reveals that rigorous evidence on the effects of agroforestry interventions on farmers’ land remains extremely limited. This finding is especially notable given the large volume of literature documenting the uptake of specific agroforestry practices and widespread promotion of agroforestry as a strategy to advance the 2030 UN SDGs. It is also somewhat surprising given the relative prevalence of impact evaluations in the related fields of agriculture and forestry.

The complexity of agroforestry poses challenges for impact evaluation. But, given the potential of agroforestry to contribute to a number of the SDGs simultaneously, there is an urgent need to address such challenges and conduct more high‐quality studies of the effects of agroforestry interventions on agricultural productivity, ecosystem services, and human wellbeing.

The most urgent need in this field is to address the gap in primary evidence. However, SR of some of the available impact studies may be useful to establish a baseline. A review of the evidence on how incentive provision and farmer capacity development interventions affect all three outcome categories would be especially useful. Such synthesis would provide insights to inform future policy and programming relating to agroforestry interventions and also present an important baseline for future research.

## 3 BACKGROUND

### 3.1 The problem, condition, or issue

The integration of trees in agriculture is widespread across the L&MICs of Africa, Asia, and Latin America. Agroforestry practices, ranging from farmer‐managed natural regeneration through to the intercropping of trees within annual crop fields and cultivation of forest gardens, are estimated to take place on nearly 50% of agricultural land in developing country regions (Zomer et al., 2014). Defined simply as “agriculture with trees” or more comprehensively as “the practice and science of the interface and interactions between agriculture and forestry, involving farmers, livestock, trees and forests at multiple scales” (World Agroforestry, 2017), agroforestry comprises an increasingly important strategy to increase farmer income and food production while advancing other social and environmental objectives.

### 3.2 The intervention

Proponents argue that agroforestry can provide basic subsistence, natural insurance, and a means to generate income and build assets for many rural households in L&MICs (Garrity et al., 2010; Miller, Muñoz‐Mora, & Christiansen, 2017). Agroforestry can also generate environmental benefits, including carbon storage, biodiversity conservation, clean water, erosion control, and soil fertility, while enhancing resilience of agricultural lands in the face of climate‐related stresses (FAO, 2013; Garrity et al., 2010; Jose, 2009; Kalaba, Chirwa, Syampungani, & Ajayi, 2010; Mbow, Smith, Skole, Duguma, & Bustamante, 2014). In addition, studies also suggest agroforestry has the potential to increase agricultural productivity (Garrity et al., 2010; Pretty & Bharucha, 2014; Sileshi, Akinnifesi, Ajayi, & Place, 2008; Waldron, Justicia, & Smith, 2015).

Given these potential benefits, agroforestry has been widely promoted in L&MICs. It is expected to play a key role in delivering the UN SDGs (United Nations, 2015; Waldron et al., 2017; World Agroforestry, 2017). Government extension agencies, NGOs, and a range of donor agencies have long provided support to agroforestry systems and practices. Since the 1992 UN Earth Summit in Rio, international aid donors have invested more than U.S. $10 billion in agroforestry projects (AidData, 2017; activity code: 31220.07) in L&MICs (Tierney et al., 2011). The largest donor, the World Bank, continues to emphasize agroforestry in its policy documents, including major commitments to ensure its agricultural investments are “climate smart” by 2020 (World Bank 2016). High‐level policy documents in many L&MICs now explicitly call for the integration of trees into farming systems (e.g., national policies of Government of India, 2014; Republic of Kenya, 2014; and Government of Malawi, 2011) and there is growing interest in promoting agroforestry as part of sustainable intensification initiatives that reconcile agricultural production with the provision of other important ecosystem services (FAO 2013; Pretty, 2018).

A large body of literature on agroforestry in L&MICs has accumulated, but systematic understanding of the effects of agroforestry on social and ecological outcomes within and across diverse contexts is missing. This lack of knowledge, in turn, constrains the ability of policymakers, practitioners, and researchers to make effective decisions relating to agroforestry programming and investments. This EGM provides such an overview. Specifically, the EGM identifies, collects, maps, and describes available high‐quality evidence on the effects of interventions promoting agroforestry on agricultural productivity, ecosystem services, and human well‐being in the L&MICs of Africa, Asia, and Latin America. It shows areas of high, low, or nonexistent evidence, as well as varying levels of robustness relative to study design.

This EGM differs from other EGMs in that we also included and describe the literature on agroforestry *practices* that may have been put in place without being promoted by any specific intervention. The motivation for this decision was twofold. First, the uptake of agroforestry practices need not rely on external interventions and, second, “adoption studies” have been especially prominent in this field.

### 3.3 How the intervention might work

There is no standardized way in which agroforestry is promoted. Agroforestry policies and programs can be shaped by a variety of factors, including the social‐ecological context in which they are implemented, the specific objectives, knowledge, and interests of the external organization and farmers involved, and the financial, technical, and material (including tree/shrub germplasm) resources available (Garrity et al., 2010). Nevertheless, we identify at least six different classes of interventions—elaborated in greater detail in Table 2—through which agroforestry is generally promoted and encouraged:

- *Farmer capacity development* through training, extension, the provision of other advisory services and technical information, demonstration sites, participatory trials, and other modes of action learning.
- *Enhancing access to tree germplasm* through the direct provision of tree seedlings/seeds and linking farmers to and/or strengthening the capacity of tree germplasm suppliers.
- *Community‐level campaigning and advocacy* encouraging large numbers of community members to plant trees on their farms and/or pursue specific agroforestry practices.
- *Incentive provision* through direct payments to farmers for planting and caring for trees on their farms and the receipt of premiums for particular agricultural commodities, for example, for shade grown coffee.
- *Market linkage facilitation* for a greater and/or more favorable integration of smallholders into tree‐product value chains.
- *Policy and institutional change* for a more enabling environment that promotes the uptake of agroforestry and/or enables its potential benefits to be better realized.

Although there is wide variation in the practices promoted, agroforestry interventions typically encourage farmers to take up several complementary practices to meet multiple social‐ecological objectives (Waldron et al., 2017). For example, planting of tree species that will generate productive uses only over the long‐term may be promoted at the same time as crops and shrubs that provide benefits in the near term. We present a classification scheme for a range of agroforestry practices in Table 1.

**Table 1:** Classification of agroforestry systems and specific practices

Agroforestry system	Specific practices	Definition
Agrisilvicultural (crops and trees)	Improved or rotational fallow	Land resting system using trees and shrubs to replenish soil fertility, sometimes in rotation with crops as in traditional shifting cultivation
	Multipurpose trees on parklands or lots (mixed trees and crops)	Scattered trees in parklands (landscapes derived from agricultural activities) or other land area or in systematic patterns on bunds, terraces, or plot/field boundaries
	Mixture of plantation crops	Combination of plantation crops in an intercropping system in alternate arrangement, including use of shade trees for cash crops
	Tree gardens	Cultivation of a mixture of several fruit and other useful trees, sometimes with the inclusion of annual crops. This arrangement is sometimes referred to as homegardens
	Alley cropping	Planting rows of trees with a companion crop grown in the alleyways between the rows
	Shelterbelts	Extended windbreak of living trees and shrubs established and maintained to protect farmlands (beyond a single farm)
Silvopastoral (pasture/animals and trees)	Multipurpose fodder trees or shrubs around farmlands (protein bank)	Production of protein‐rich tree fodder on farm/rangelands
	Living fences and shelterbelts	Trees as fences around plots and/or an extended windbreak of living trees and shrubs established and maintained to protect farmlands and provide fodder
	Integrated production of animal/dairy and wood products	Production of animal/dairy and wood products within the same land area
	Trees/shrubs on pasture	Trees scattered irregularly or arranged according to some systematic pattern
Agrosilvopastoral (crops, pasture/animals, and trees)	Integrated production of animals (meat and dairy), crops, and wood/fuelwood	Production of crops, animal/dairy, and wood products within the same land area, including around homesteads
	Woody hedgerows for browse, green manure, soil conservation	Multipurpose woody hedgerows for browse, mulch, green manure, soil conservation, and so forth
	Wooded pasture products	Land covered with grasses and other herbaceous species, and with woody species
Agroforestry including insects/fish	Entomoforestry	The combination of trees and insects (e.g., bees for honey and trees)
	Aqua‐silvo‐fishery	Trees lining fish ponds, tree leaves being used as “forage” for fish

*Note*: The broad systems listed in this table are based on Nair (1985) and specific interventions derived from Nair (1985, 1993), Sinclair (1999), and Atangana et al. (2014). Definitions are drawn from Huxley et al. (1997).

**Figure 1:**
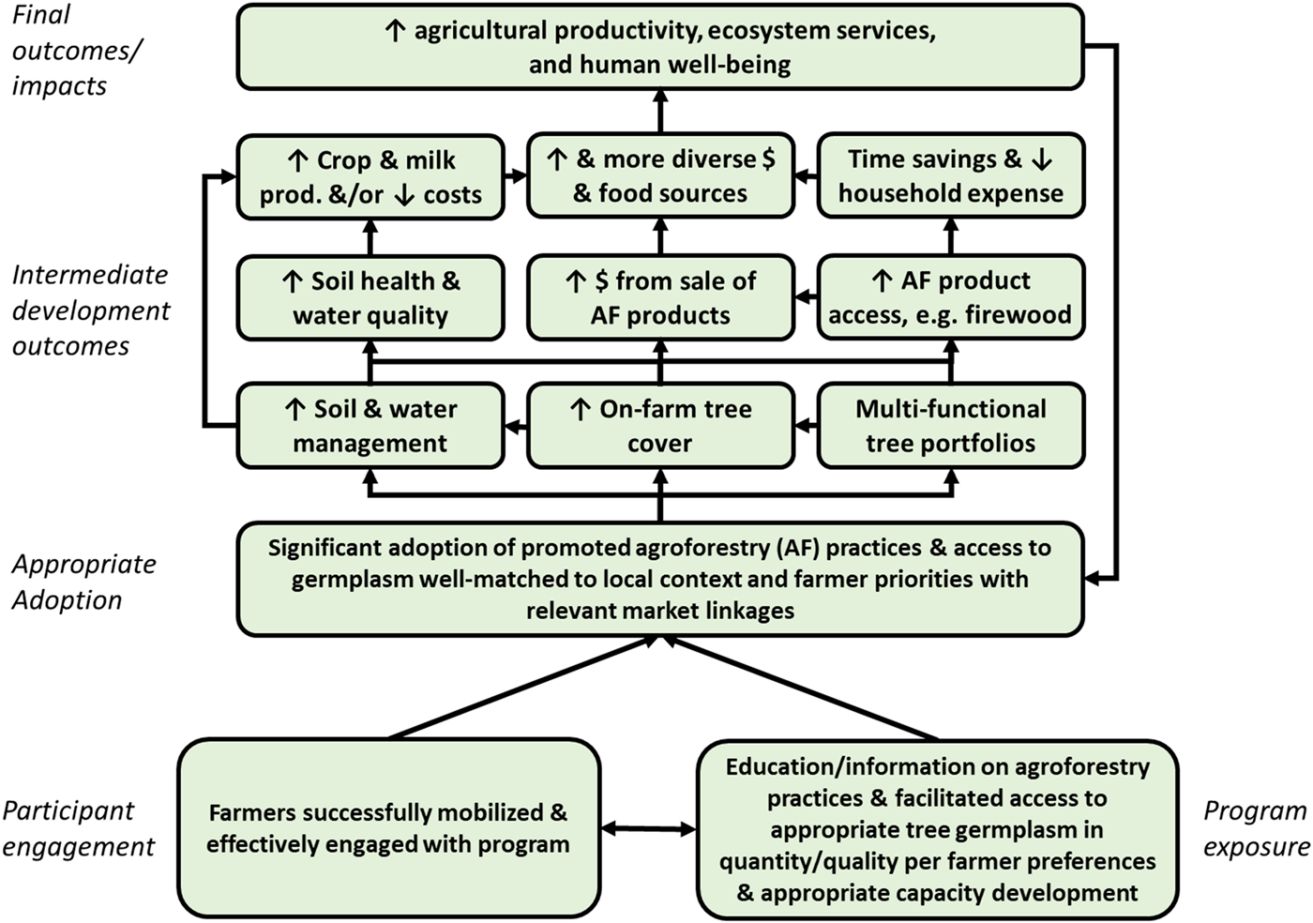
Illustrative theory of change for an AF intervention. AF, agroforestry

Figure 1 presents a simplified and generic theory of change which may underlie an agroforestry intervention (either explicitly or implicitly). The first required step is successful mobilization and engagement of farmers. The second step represents a given intervention, such as farmer capacity development or facilitating access to appropriate tree germplasm. At least the first and, in many cases, both are required for significant and appropriate adoption of the promoted agroforestry practices and/or tree germplasm. Following such adoption, several intermediary outcomes are then expected. For example, farmers may see improved soil health and other ecosystem services, such as water filtration, that then increase crop productivity or reduce production costs and, therefore, increase returns. Some participants in the intervention may find that increased use and availability of tree/shrub fodder leads to increases in milk and other livestock production and returns. Selling other agroforestry products such as timber, firewood, and fruit, is also expected to increase and diversify income and food sources (Mbow, Van Noordwijk, et al., 2014; Sharma et al., 2016; Waldron et al., 2017). These changes may have differential effects depending on gender. Together, these intermediate outcomes are expected to interact together to bolster household resilience to shocks, as well as overall household income food and nutritional security. These positive benefits—and the broader context in which this stylized theory of change is embedded—will then affect further household investment in agroforestry.

### 3.4 Why it is important to do the review

As described above, agroforestry systems and practices are widespread across L&MICs and have increasingly been seen as a solution for boosting food security, addressing environmental degradation, and contributing to a range of other development policy objectives (Garrity et al., 2010; Waldron et al., 2017). Nevertheless, financing and effective promotion of agroforestry and other nonmainstream agricultural approaches remains limited in many contexts (DeLonge, Miles, & Carlisle, 2016; Horlings & Marsden, 2011; IPES‐Food 2016).

Instead, high‐input, mechanized approaches to agriculture predominate. Over the past half century, these approaches have become conventional, leading to major increases in yields and helping to feed much of the world's population (Iaastd, 2009; Pretty & Bharucha, 2014; The Government Office for Science, 2011). However, these benefits have brought with them sometimes steep social and environmental costs, including biodiversity loss, climate change, land degradation, water pollution, and negative effects on human health (Brawn, 2017; Horrigan, Lawrence, & Walker, 2002; Iaastd 2009; Maxwell, Fuller, Brooks, & Watson, 2016; Pretty & Bharucha, 2014).

Farmers, consumers, and policymakers increasingly recognize these costs and seek viable alternatives that can simultaneously address food security concerns while delivering other social and environmental benefits. Agroforestry represents one such potential alternative, but there is an important need to systematically identify what kinds of interventions and practices have worked to deliver these benefits and understand potential trade‐offs involved. Evidence on the effectiveness of agroforestry is, therefore, needed to inform broader debates and investment decisions relating to sustainable agricultural intensification.

Despite the long history of agroforestry systems and practices, agroforestry as a specific science and specific policy domain emerged only in the 1960s and 1970s. National governments, NGOs, research organizations, and aid agencies alike began to embrace the idea and to develop, test, and support a wide range of agroforestry practices (Nair, 1993). As the field has matured, a substantial literature on the adoption and impacts of agroforestry practices in L&MICs has developed. However, syntheses of evidence of what agroforestry practices have been effective, under what circumstances, and why remains lacking.

Recent systematic maps (SMs) and SRs have begun to shed light on the effects of agroforestry practices on specific outcomes, such as agricultural productivity and ecosystem service provision (Reed et al., 2017; Rosenstock et al., 2016; Thorn et al., 2015). Cheng et al. (2019) examine the impacts of forestry and agroforestry interventions on poverty. The recently published SR by Reed et al. (2017) synthesizes existing evidence on the indirect effects that forest‐ and tree‐related ecosystem services have had on food production in the tropics. Two recent EGMs related to forests (Puri, Nath, Bhatia, & Glew, 2016; Snilstveit et al., 2016) include agroforestry, with some attention to existing evidence on effects on environmental and social outcomes in L&MICs. These reviews provided valuable information for this EGM, which is broader in scope geographically and in outcomes considered. As detailed below, this EGM includes all L&MICs, not just tropical ones, and direct and indirect effects of agroforestry interventions on a range of outcomes. We are aware of no EGM, SM, or SR that summarizes empirical studies on the causal effects of agroforestry interventions in L&MICs, particularly outside the context of tightly controlled, research station‐based experimental trials.

There are two primary audiences for this EGM. First, we expect that researchers on agroforestry and broader sustainability issues will use the results to inform further investigations on these topics, including new empirical research, as well as SRs of specific linkages and further evidence synthesis. Results should be of wide interest to researchers in a range of institutions, from CGIAR centers to universities. The second main anticipated audience is decision‐makers for whom agroforestry is already or potentially of interest. This includes relevant ministries and programs in governments and donor agencies, as well as NGO and other advocacy and implementing organization staff.

## 4 OBJECTIVES

### 4.1 The problem, condition, or issue

The overall aim of this EGM is to identify, map, and describe existing evidence on the effects of agroforestry interventions on agricultural productivity, ecosystem services and human well‐being in L&MICs. The results will inform the scope of a planned SR on this topic.

In doing so, it addresses the following research questions:

(1) What are the extent and characteristics of empirical evidence on the effects of agroforestry interventions and practices on agricultural productivity, ecosystem services, and human well‐being in L&MICs?
(2) What are the major gaps in the primary evidence base?
(3) What are the agroforestry intervention/practice and outcome areas with potential for evidence synthesis?

## 5 METHODS

Our framework follows standard practice for EGMs (Snilstveit, Vojtkova, Bhavsar, & Gaarder, 2013), with rows in a matrix representing interventions and columns outcomes. Below we detail these two dimensions of the matrix as well as describe the PICO component that we examined.

### 5.1 Criteria for considering studies for this review

To identify the effect of an intervention or practice, a study needs to include both adopters (or program participants) and a comparator. A comparator is defined as a farm or household that does not adopt a given practice identified in Table 1, or is not exposed to a specific agroforestry intervention. Specifically, eligible comparisons included land or households where agroforestry was not practiced or promoted but another land use was in place (e.g., agriculture, primary forest, or secondary forest/forest plantation). For observational studies, a farm or household before agroforestry promotion or adoption of a given agroforestry practice began was also an eligible comparator.

#### 5.1.1 Types of participants

The population of interest was farms and those that live and farm on them in L&MICs using a system that falls within the definition of agroforestry.

#### 5.1.2 Types of interventions

The overall intervention category for our EGM is “agroforestry” defined as “a collective name for land‐use systems and technologies where woody perennials (trees, shrubs, palms, bamboos, etc.) are deliberately used on the same land‐management units as agricultural crops and/or animals, in some form of spatial arrangement or temporal sequence” (Nair, 1993). In the field of agroforestry, there are multiple strands of literature, including studies of the impacts of specific agroforestry practices and systems and studies of the impacts of specific interventions designed to spur the adoption of agroforestry to yield more distal social‐ecological impacts. The “intervention” axis in this EGM therefore includes both categories.

To capture the wide diversity of practices that might fall under this definition and present them in a coherent way, we subdivided agroforestry into the practice types listed in Table 1. This set of practice types is based on the classification system proposed by Nair (1985, 1993) and updated by Sinclair (1999), Torquebiau (2000), and Atangana et al. (2014).

From a policy perspective, it is especially useful to know what kinds of interventions might most effectively promote agroforestry practices to yield desired social‐ecological outcomes. The EGM, therefore, also includes studies that examine specific types of interventions designed to promote agroforestry. The intervention types are summarized in Table 2.

**Table 2:** Classification of interventions to promote agroforestry

Intervention type	Description and examples
Farmer capacity development	Efforts focus on enhancing farmer knowledge and/or skills relevant to agroforestry practice, for example, setting up and managing tree nurseries, tree planting and management techniques, and seed collection and propagation. Such interventions can involve the provision of training, extension, and other advisory services, and specific technical information, as well as the setting up of demonstration sites, running of participatory trials, and other modes of participatory action learning
Enhancing access to tree germplasm	Efforts to facilitate farmer access to quality and desired tree/shrub seedlings/seeds required to pursue prioritized agroforestry practices. Such interventions often entail the direct provision of seedlings/seeds to farmers but can also involve linking farmers to relevant suppliers and/or enhancing the ability of existing or new suppliers to supply participating farmers with quality and desired tree germplasm
Community‐level campaigning and advocacy	Interventions of this type can also involve the provision of information about the benefits of trees and agroforestry and/or the provision tree seedlings/seeds but is distinct from the first two types. The main objective is to motivate, including through social pressure, community members to plant trees on their farms and/or pursue specific agroforestry practices. Campaigning and advocacy may be done through radio and/or community meetings, speeches, and drama and may involve a mass community effort to plant trees, for example, on a specific day of the year
Incentive provision	Interventions of this type seek to motivate farmers to plant trees and practice agroforestry through the provision of incentives. Examples include paying farmers for planting and caring for trees on their farms in exchange for desired ecosystem services (e.g., carbon sequestration) and buyers offering premiums to farmers for agricultural commodities produced under certain conditions (e.g., via certification schemes for products such as shade grown organic coffee)
Market linkage facilitation	Interventions of this type focus on efforts to enhance potential returns from agroforestry to encourage adoption. This could be through linking producers to and/or brokering new and/or improving existing contractual arrangements with buyers. Other examples include the collective marketing of agroforestry products and/or interventions to stimulate demand for a given agroforestry product, for example, Baobab fruit
Institutional and policy change	Interventions of this type involve reforming and/or putting in place new policies, laws, regulations, and institutions more broadly to facilitate greater uptake of and benefits from agroforestry. Such efforts are designed to address existing policy and institutional constraints such as, for example, prevailing forestry regulations—designed for forest management areas—that may frustrate smallholder efforts to grow particular high‐return tree species or insecure land tenure that may similarly deter long‐term investments in tree planting

We present the main matrices of interventions and practices in two ways: (a) a simplified typology of interventions and practices using the broad agroforestry systems listed in Table 1 and (b) a more detailed version with the specific interventions and practices listed in Table 1.

#### 5.1.3 Types of outcome measures

The columns of the EGM matrix comprise three broad outcome categories: (a) agricultural productivity, (b) ecosystem services, and (c) human well‐being. Studies that focused exclusively on the adoption of a particular agroforestry technique or species without reference to effects on outcomes were excluded.

Specific outcome categories under agricultural productivity comprise factor productivity, including yield, and profitability. Ecosystem services outcomes were first classified under three broad categories: (a) provisioning, (b) regulation and maintenance, and (c) cultural services. Outcomes were then further divided into a number of specific categories following the Common International Classification of Ecosystem Services (CICES) developed by the European Environmental Agency (Haines‐Young & Potschin, 2012) and presented in Table 3. CICES builds from the seminal Millennium Ecosystem Assessment (2005), The Economics of Ecosystems and Biodiversity (Kumar, 2012), and other ecosystem services classification schemes.

**Table 3:** Classification of ecosystem services outcomes in broad and specific categories

Broad category	Specific category	Examples
Provisioning	Energy	Biomass‐based energy sources (plant and animal)
		Mechanical energy (animal‐based)
	Materials	Biomass (e.g., fiber and other materials from plants, and animals for direct use or processing)
		Water (surface or groundwater for nondrinking purposes)
	Nutrition	Biomass (e.g., cultivated crops, reared animals and their outputs, wild plants and animals and their outputs, etc.)
		Water (e.g., surface or groundwater for drinking)
Regulation and maintenance	Mediation of waste, toxics, and other nuisances	Filtration/sequestration/storage/accumulation/mediation of smell/noise/visual impacts
		Weed and pest control
	Mediation of flows	Mass stabilization and control of erosion rates
		Hydrological cycle and water flow maintenance
		Flood and storm protection
		Ventilation and transpiration
	Maintenance of physical, chemical, biological conditions	Lifecycle maintenance, habitat, and gene pool protection (pollination and seed dispersal, maintaining nursery populations and habitats)
		Pest and disease control
		Soil formation and composition
		Water conditions
		Atmospheric composition and climate regulation
Cultural	Physical and intellectual interactions with environmental settings	Physical and experiential interactions (use of plants and animals)
		Intellectual and representative interactions (scientific, education, heritage/cultural, esthetic, etc.)
	Spiritual, symbolic, and other interactions with environmental settings	Spiritual and/or emblematic (symbolic, sacred, and religious use of plants and animals)
		Other cultural outputs (existence, bequest of plants and animals)

*Note*: Specific categories divide each broad ecosystem services category into main types of output or process (Haines‐Young & Potschin, 2012).

For human well‐being outcomes, we adapted the classification published in McKinnon et al. (2016) to identify a set of key policy‐relevant domains of human well‐being (Table 4). Based on likely policy interest and goals typically articulated by proponents of agroforestry, we focused on five dimensions of human well‐being: (a) income and household expenditure, (b) housing and material assets, (c) food security and nutrition, (d) health, and (e) cultural and subjective well‐being. We also included the category of “other” which may group some studies focusing on additional dimensions of human well‐being identified in McKinnon et al. (2016).

**Table 4:** Domains and definitions of human well‐being outcomes

Domain	Definition
Income and household expenditure	Total household income and expenditure, farm and nonfarm income, employment, employment opportunities, wealth, poverty, savings, payments, loans
Housing and material assets	Shelter, assets owned, access and availability of fuel and basic infrastructure (electricity, water, telecommunications, and transportation)
Food security and nutrition	Physical and economic access to sufficient, safe, and nutritious food that meets dietary needs and food preferences for an active and healthy life (FAO). Usually measured using food consumption, expenditure, prevalence of undernourishment, and nutritional status
Health	Physical health, longevity/life expectancy, maternal health, child health, access to health care, occurrence of diseases, mental health
Cultural and subjective well‐being	Measures of happiness, quality of life, cultural, societal, and traditional values of nature, sense of home, cultural identity, and heritage, spiritual or religious beliefs and/or values
Other	For example informal education (i.e., transfer of knowledge and skills), social relations (i.e., interactions between individuals and within and/or between groups), governance (i.e., structures and processes for decision making including both formal and informal rules), land and resource security, freedom of choice and action (i.e., ability to pursue what one values doing and being), adaptive capacity and resilience (i.e., ability to cope with perturbations and take advantage of new opportunities due to social and environmental change, especially climate impacts)

*Note*: Human well‐being domains and definitions adapted from McKinnon et al. (2016).

Abbreviation: FAO, Food Agriculture Organization.

As for intervention types, we present the three outcomes in the EGM main matrix in two ways: (a) a simplified typology of broad outcome categories and (b) a more detailed version with the specific outcome categories.

### 5.2 Search methods for identification of studies

This EGM includes three kinds of studies: (a) quantitative impact evaluations, (b) SRs, and (c) observational studies on the effects of agroforestry interventions and practices. Consistent with established Campbell Collaboration guidance for EGMs, our discussion of agroforestry interventions only includes the first two types of studies.

*Impact evaluations* are studies that measure changes that occur due to an intervention. Such studies use experimental or quasiexperimental designs to estimate counterfactuals so that changes in a given outcome can be attributed to a specific intervention (Cook, Campbell, & Shadish, 2002). We include the following types of quantitative impact evaluation studies:

- Studies where participants are randomly assigned to treatment and comparison group (experimental study designs).
- Studies where assignment to treatment and comparison groups is based on other known allocation rules, including a threshold on a continuous variable (regression discontinuity designs) or exogenous geographical variation in the treatment allocation (natural experiments).
- Studies with nonrandom assignment to treatment and comparison group that include pre‐ and posttest measures of the outcome variables of interest to ensure equity between groups on the baseline measure, and that use appropriate methods to control for selection bias and confounding. Such methods include statistical matching (e.g., propensity score matching [PSM], or covariate matching), regression adjustment (e.g., difference‐in‐differences, fixed effects regression, single difference regression analysis, instrumental variables, endogenous switching regression, and “Heckman” selection models).
- Studies with nonrandom assignment to treatment and comparison group that include posttest measures of the outcome variables of interest only and use appropriate methods to control for selection bias and confounding, as above.

Ideally, studies would include baseline and postintervention data, but given the small number of studies meeting this criterion, we included studies with just postintervention outcome data, only if they use some method to control for selection bias and potential confounding factors.

We also included *systematic reviews* and evidence synthesis efforts (e.g., SMs, EGMs) that describe methods used for search, data collection, and synthesis as per the standardized checklist highlighted in Snilstveit et al. (2017) for appraising SRs. Literature reviews that did not describe methods used for search, data collection, and synthesis were not included.

Finally, we also included studies on the outcomes of agroforestry practices (observational or experimental) and of agroforestry interventions (observational). *Observational studies* of agroforestry interventions and practices could be quantitative and needed to include at least one comparison as described above (e.g., before/after; adopter group/nonadopter group). However, these studies do not account for nonrandom assignment between treatment (agroforestry practice) and control (nonagroforestry practice) groups. Observational studies include any type of correlational studies, that is, where a regression equation is estimated, with outcomes as the dependent variable and an agroforestry practice as an explanatory variable, or a comparison of means of outcomes between the practice of interest (agroforestry) and a control (conventional agriculture or forestry).

### 5.3 Data collection and analysis

#### 5.3.1 Selection of studies

Studies on agroforestry practices, without a specific intervention, could be experimental (on‐farm trial) or observational. These studies evaluate the difference in outcomes between practicing agroforestry or practicing an alternative land use (agriculture, forestry) and were included because agroforestry is widely practiced as a traditional land use system without the support of external interventions, and the relative impacts of these practices may of interest for future policy direction.

The types of studies we considered all included an assessment of the outcomes of agroforestry interventions and practices against a comparable control case (conventional agriculture or forestry). We note, however, that differences between included study designs have implications on the quality and risk of bias for each study. The types of study design listed in order of highest to lowest risk of bias are: correlational studies, quasiexperimental impact evaluations, and randomized experimental designs.

We excluded theoretical or modeling studies (unless they include a relevant empirical example with design that meets inclusion criteria), editorials and commentaries, and field trials that did not take place on farmer land (e.g., that were conducted at agricultural research stations or universities).

Field trials in agroforestry are designed to test the effects of experimental treatments or other variables on crop yield or other outcomes of interest in conditions similar to the actual growing conditions experienced by farmers who may adopt the treatment. While impact evaluations measure the changes due to an intervention, field trials measure the changes due to a practice. As for agronomy more generally, field trials can be divided into three types: (a) researcher managed and researcher implemented, (b) researcher managed and farmer implemented, and (c) farmer managed and farmer implemented. We included field trials only if they were implemented on a farmer's land, included an experimental research design, and described the effects of an intervention, technique, or practice on an outcome category relevant to the current study. Other kinds of field trials were excluded given the focus of our research on the effectiveness of agroforestry in “real world” on‐farm settings. We also excluded such field trials due to the large volume of studies and because field trials typically address questions of efficacy rather than effectiveness.

The methodology we used in conducting this EGM is detailed in Miller, Ordonez, et al. (2017). We summarize it here and note some small changes from the protocol. We defined a search strategy and the databases to be searched (see Appendix A for further detail), as well as a strategy for searching gray literature, based on Haddaway et al. (2017). We tested our search strategy against a test list of studies known by the authors to be relevant to the current study. Studies from the year 2000 to June 30, 2017 were included in the search. We began the study period in 2000 given that year marked the start of the Millennium Development Goals, which presaged the current SDGs, and that agroforestry had gained significant momentum by then in the wake of the 1992 UN Earth Summit in Rio. The search was carried out in October 2017, but only studies published before July 1, 2017 were considered. The results from the searches were uploaded to EPPI‐Reviewer v4. Resource constraints meant we only included studies published in English.

We imported the records from academic databases into our data management software (EPPI‐Reviewer 4), and we used the built‐in tool to aid in removing duplicates. The gray literature was imported into and managed in Microsoft Excel due to reference format incompatibility with EPPI‐Reviewer 4. We screened the records at the title and abstract level, excluding studies which did not meet our criteria for study country, publication year, study type, and relevant agroforestry practice or intervention.

The review process consisted of 14 reviewers. All the reviewers were trained by the project leads (D. C. M. and K. B.) and research coordinators (P. J. O. and S. E. B.). The title and abstract stage of the review process included 11 reviewers. To ensure inter‐rater reliability, each reviewer was given two samples of 30 studies for classification. Results from one of the lead researchers was used as the standard for classification and a *κ* statistic was used as a measure of agreement between reviewers (Cohen, 1960). This statistic was calculated for each reviewer against the standard classification. At least a 70% agreement was required for all reviewers. If the initial sample did not yield the required agreement, reviewers would discuss their responses with a project lead and retake the test until the required agreement level was reached. Once the review process started, if a reviewer was unsure about the inclusion of a given study, the reviewer had the option to mark it for a second opinion. The research leads and coordinators made inclusion decisions in such cases. In addition, these same reviewers performed a second title and abstract screening of all studies marked for inclusion, at which point some additional studies were excluded that were found not to meet the inclusion criteria.

The full team of reviewers then screened remaining studies at the full text level. At this stage, reviewers also had the option to mark studies for second opinion, with the lead researchers making final determinations as in the previous stage. Throughout the screening process, the research team met regularly to discuss any issues or inconsistencies and spot‐checking was done by the lead researchers/research coordinators.

#### 5.3.2 Data extraction and management

We used a standardized data extraction form (presented in Appendix B) to extract descriptive data from all studies meeting the eligibility criteria. A more detailed codebook describing the scope of each component of the data extraction form was also created and is available upon request. This standard was followed by all coders conducting data extraction. Finally, the lead researchers (authors P. J. O. and S. E. B.) checked the data extraction for all included studies to ensure consistency and completeness. Any studies identified as reviews were screened based on the standardized checklist highlighted in Snilstveit et al. (2017), and we conducted a study critical appraisal of the included SRs per the same checklist. We have attached the checklist used for screening and appraisal in Appendix A. We used a standardized checklist to assess our confidence in the findings of each SR (Snilstveit, Eyers, Bhavsar, Gallagher, & Stevenson, 2014). The confidence ratings do not appraise the studies included in a review, but rather the methodology and reporting of the review.

In our data extraction, we noted any reference to equity in the included studies. Equity focus is defined as the extent to which an intervention or analysis focuses on specific disadvantaged populations. We aimed to identify how and to what extent the included studied considered equity in their approach. We used the PROGRESS framework (O'Neill et al., 2014) to consider potentially disadvantaged groups in the included studies. Key dimensions of equity that we considered were gender, race/ethnicity, socioeconomic level, and literacy/educational level. We assessed the extent to which each study addresses equity, by describing any intervention focus on specific social groups, examining equity as an outcome, or reporting on differential impacts across subpopulations.

#### 5.3.3 EGM structure

The main results of the EGM is presented as a visual representation of the existing evidence in matrix form Snilstveit et al. (2017), with rows representing the different categories of agroforestry practices (or interventions), and columns representing the outcomes under the three categories included. In addition, the characteristics of the included studies is analyzed and presented using descriptive statistics, with graphical representation of the most important aspects of the data.

Resource constraints meant we did not include any studies in a language other than English. Two other changes from our original protocol were to exclude field trials (given the extensive volume of studies and their unclear linkage to interventions) unless they occurred on farmers’ land and to include a comparator for practices other than agroforestry on a given piece of land (e.g., agriculture, primary forest, or secondary forest/forest plantation). This latter change was made early in the process when we realized we risked excluding studies that made such nonagroforestry comparisons. Screening prior to that period was redone to ensure consistency through the process.

#### 5.3.4 Methodological limitations

This EGM differs from other Campbell Collaboration EGMs in that it not only examines evidence on the impacts of interventions that promote agroforestry but also includes studies on the impacts of specific agroforestry practices. This is a strength of our EGM, but also presents a potential limitation, given that many studies of practices are correlation studies, with no experimental studies and very few quasiexperimental studies. As such, the evidence they present does not fully control for unobserved variables that may be correlated with both the specific agroforestry practices and the measured outcomes.

We only included studies in English, which can limit the scope of our results by not including relevant studies in other languages. For example, our study has likely missed important evidence described in French, Mandarin Chinese, Portuguese, and Spanish, among other languages. Similarly, given that the practice of agroforestry has different names in different places, is possible that we missed a relevant term in our search strategy, even though the terms we used were developed in consultation with a search specialist and our advisory team, which included several experts in this field.

## 6 RESULTS

This section reports and discusses the 396 studies identified from our search and screening process for this EGM. We describe the search and screening process, discuss the characteristics and trends of the evidence base, and examine the body of literature for concentrations and gaps in the evidence.

The number of studies shown in each distribution chart refers to the total number of studies falling under each domain presented. Individual studies may be classified under multiple domains. For instance, if a study examines the impacts of multiple practices, that study would add to the count for each practice associated with that paper. The sum of studies for each figure may therefore be greater than the number of unique studies associated with that figure.

### 6.1 Description of studies

#### 6.1.1 Results of the search

**Figure 2:**
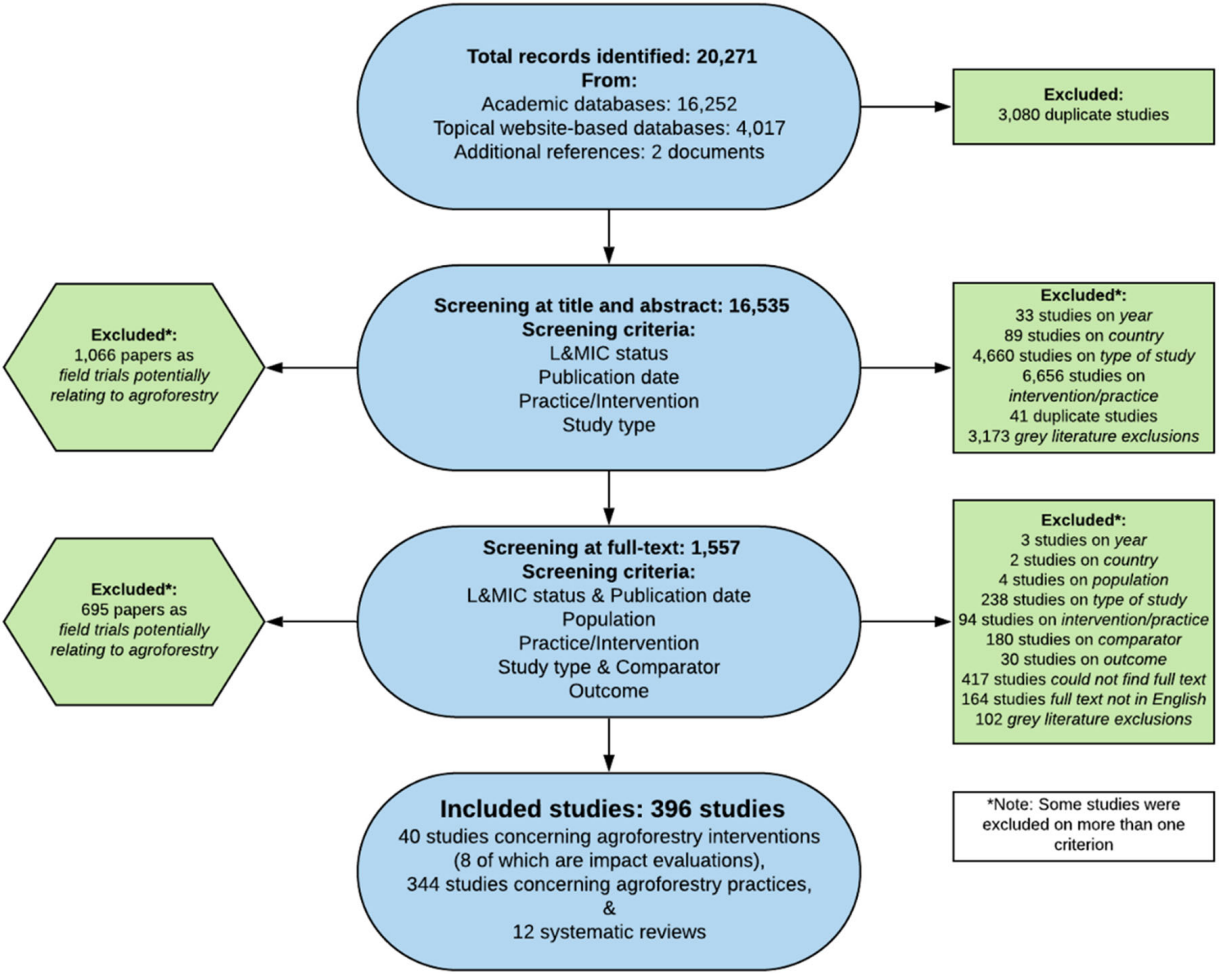
PRISMA flow diagram. PRISMA, preferred reporting items for systematic reviews and meta‐analyses

Figure 2 provides an overview of the results of the search and screening process to identify studies for inclusion. The search returned 20,271 records, with 16,535 studies remaining for screening at title and abstract after duplicate removal.

As with other areas of social science (Waddington et al., 2012), many studies used titles or abstracts that did not clearly indicate the research topic explored, making it difficult to determine whether the paper met the inclusion criteria. As a result, 1,557 studies, a relatively large number of records, had to reviewed at full text. Of these, 396 met the inclusion criteria for the EGM. The main reasons for exclusion were lack of relevant intervention/practice (*n* = 6,750) and type of study (*n* = 4,898). The relatively low number of remaining studies (*n* = 963) were excluded for other reasons. Only 11 of the 4,017 studies identified from the grey literature sources were included in the final EGM.

Due to the large number of studies screened at full text, we do not provide a full list of excluded studies here, but this list is available upon request. In addition to our exclusions, we also identified approximately 1,700 studies that appeared to be field trials of different agroforestry practices/techniques. These studies were excluded and not reviewed further. However, it may be useful to review this literature in more detail in a future study to identify practices that appear efficacious.

Of the 396 included studies, 344 studies present empirical evidence on the impacts of agroforestry practices without a specific intervention, 40 report empirical evidence on the impacts of agroforestry interventions, and 12 studies are SRs. Of the 40 studies that examined the impacts of specific agroforestry interventions, only eight used quantitative impact evaluation methods. The other 32 intervention studies measured the impacts of an agroforestry intervention but did not use experimental or quasiexperimental methods. We did not identify any ongoing SRs or primary studies. A full list of included studies and the full data extraction record sheet are provided as Supporting Information.

### 6.2 Characteristics and trends on intervention impact evaluations

**Figure 3:**
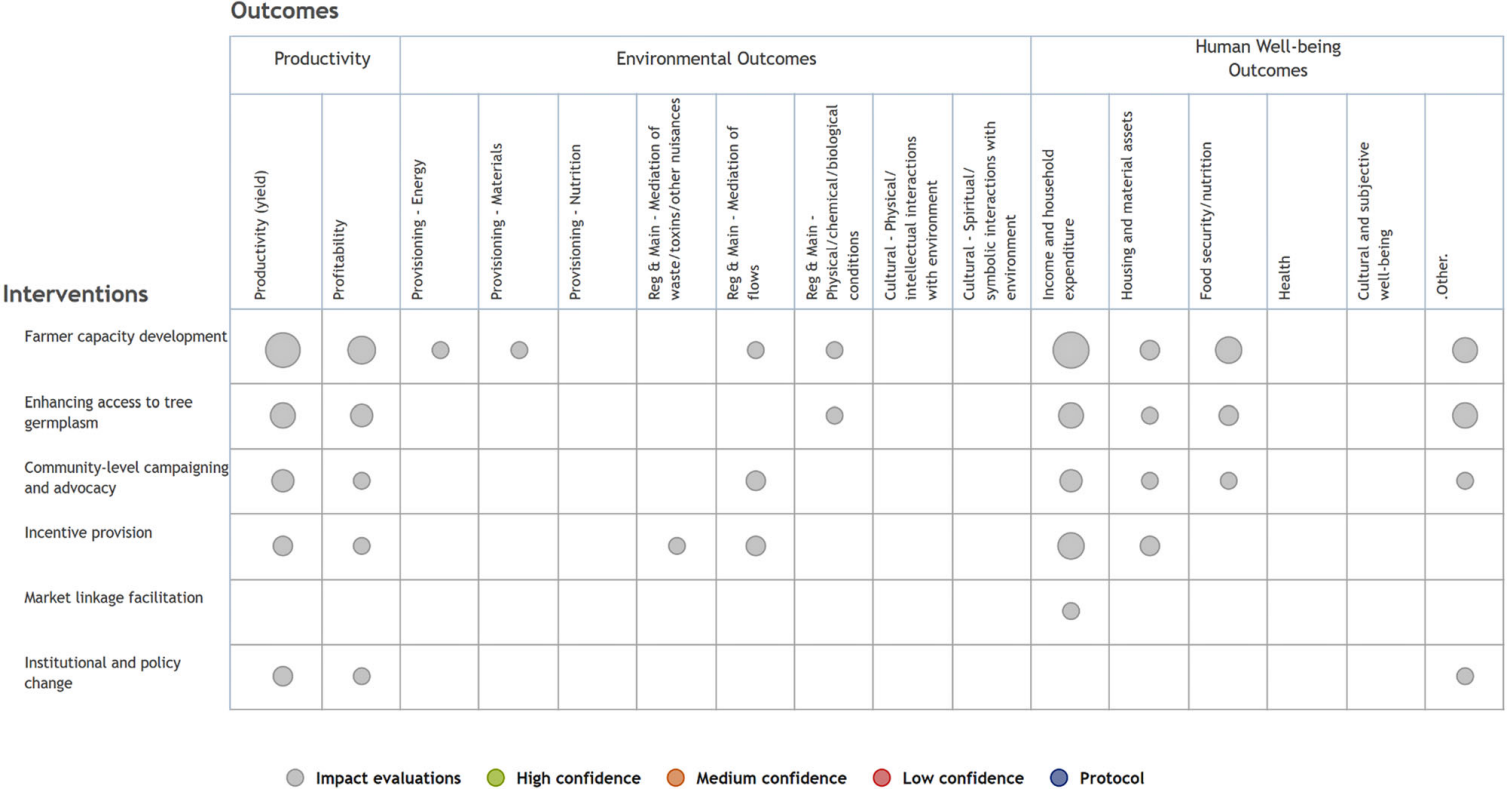
Agroforestry evidence and gap map (3ie format)

This section of the EGM covers the subset of studies that assessed the impact of agroforestry *interventions*. As mentioned in earlier, we identified six different intervention types that promote and support the use of agroforestry. These are detailed in Table 2 and shown in Figure 3. In total, we found 40 intervention‐focused studies, including eight impact evaluations using experimental or quasiexperimental approaches and 32 studies using nonrandomized approaches for evaluation. Each intervention study included mention of at least one agroforestry intervention and at least one relevant outcome. The resulting EGM is presented in Figure 3. We note that there are two main reasons linkages may have little or no evidence: (a) the linkage is of research and policy interest but has not been well studied, or (b) the linkage is not of significant research and policy interest, including cases where the practice or intervention does not link logically with a given outcome, and therefore has not been investigated.

#### 6.2.1 Experimental/quasiexperimental impact evaluation studies

We identified eight studies evaluating the impact of agroforestry interventions. All of the studies adopted quasiexperimental methods, with no studies using an experimental design. Table 5 presents basic descriptive information on these eight studies.

**Table 5:** Description of intervention studies using experimental or quasiexperimental methods

Title	Publication year	Country region	Intervention(s)^a^	Practices	Method of analysis	Outcomes	Equity measure	References
Adoption of agroforestry and the impact on household food security among farmers in Malawi	2017	Malawi Sub‐Saharan Africa	Farmer capacity development Enhancing access to tree germplasm	Agrisilvicultural trees integrated in crop fields	Endogenous switching regression	Productivity (yield) Profitability Income and household expenditure Food security and nutrition	Gender Socioeconomic level Literacy/educational level	Coulibaly et al. (2017)
Agroforestry extension and dietary diversity—an analysis of the importance of fruit and vegetable consumption in West Pokot, Kenya	2016	Kenya Sub‐Saharan Africa	Farmer capacity development Enhancing access to tree germplasm	Agrosilvopastoral integrated production of animals, crops, and wood	Heckman two‐stage probit regression	Food security and nutrition	None	Bostedt et al. (2016)
Environmental‐economic benefits and trade‐offs on sustainably certified coffee farms	2017	Nicaragua Latin America and the Caribbean	Incentive provision	Agrisilvicultural trees integrated in crop fields	Propensity score matching	Reg & Main—physical, chemical, biological conditions Reg & Main—mediation of flows Reg & Main—mediation of waste, toxics and other nuisances Income and household expenditure Productivity (yield)	Literacy/educational level	Haggar et al. (2017)
Estimating the causal effect of improved fallows on farmer welfare using robust identification strategies in Chongwe, Zambia	2013	Zambia Sub‐Saharan Africa	Farmer capacity development	Agrisilvicultural improved or rotational fallow	Propensity score matching Endogenous switching regression	Productivity (yield) Profitability Income and household expenditure Food security and nutrition	None	Kuntashula and Mungatana (2013)
Evaluation of the permanence of land use change induced by payments for environmental services in Quindio, Colombia	2016	Colombia Latin America and the Caribbean	Incentive provision	Silvopastoral trees/shrubs on pasture	Difference‐in‐differences	Reg & Main—Physical, chemical, biological conditions	Socioeconomic level	Pagiola et al. (2016)
Impacts of the Hutan Kamasyarakatan social forestry program in the Sumberjaya watershed, West Lampung district of Sumatra, Indonesia	2008	Indonesia East Asia and Pacific	Incentive provision	Agrisilvicultural trees integrated in crop fields	Propensity score matching Instrumental variables	Housing and material assets Income and household expenditure	None	Pender et al. (2008)
Performance of an agro‐forestry based payments‐for‐environmental‐services project in Mozambique: a household level analysis	2011	Mozambique Sub‐Saharan Africa	Incentive provision	Agrisilvicultural trees integrated with plantation crops	Propensity score matching	Productivity (yield) Housing and material assets Income and household expenditure	Gender Socioeconomic level Literacy/educational level	Hegde and Bull (2011)
The impact of agroforestry‐based soil fertility replenishment practices on the poor in western Kenya	2005	Kenya Sub‐Saharan Africa	Farmer capacity development Community‐level campaigning and advocacy	Agrisilvicultural improved or rotational fallow	Instrumental variables	Productivity (yield) Food security and nutrition Income and household expenditure Housing and material assets	Gender Socioeconomic level Race/ethnicity Literacy/educational level	Place et al. (2005)

^a^ In cases where the study examined interventions with multiple components more than one intervention type is listed.

The most studied interventions were farmer incentive provision (*n* = 4, 50%), which refers to any intervention that seeks to motivate farmers to plant trees and practice agroforestry through the provision of incentives (see definitions in Table 2) and farmer capacity development (*n* = 4, 50%), which refers to efforts focused on enhancing farmer knowledge and/or skills relevant to agroforestry practice.^1^ Importantly, there were no impact evaluations of two intervention types: market linkage facilitation and institutional and policy change.

Nearly all the agroforestry practices promoted in the intervention studies were agrisilvicultural (in *n* = 6, 75%). Table 5 shows the specific practices that were promoted, with trees integrated in crop fields (38%) followed by improved or rotational fallow (25%) the two most frequently promoted. The intervention studies specified the practices promoted, so there were no “general” agroforestry practices mentioned. However, this group of studies did not examine less prevalent practices such as agroforestry with insects or fish. These intervention studies were conducted from 2005 to 2017, with the year 2017 with the highest number of studies (*n* = 2). In some years no impact evaluation studies on this topic were published. All included intervention studies were published in peer‐reviewed journals (*n* = 7) except for one, which was an organization report.

Ecosystem services was the least frequent outcome category (*n* = 2, 25%), and the most frequent one was human well‐being outcomes (*n* = 6, 75%). Agricultural productivity was evaluated for half of the studies (*n* = 4, 50%). For specific outcomes, income and household expenditure was the most common outcome (*n* = 5, 63%) followed by agricultural productivity (*n* = 4, 50%). When looking at the combination of interventions and outcomes, the most studied linkages were studies focused on incentive provision and farmer capacity development with human well‐being and agricultural productivity outcomes.

**Figure 4:**
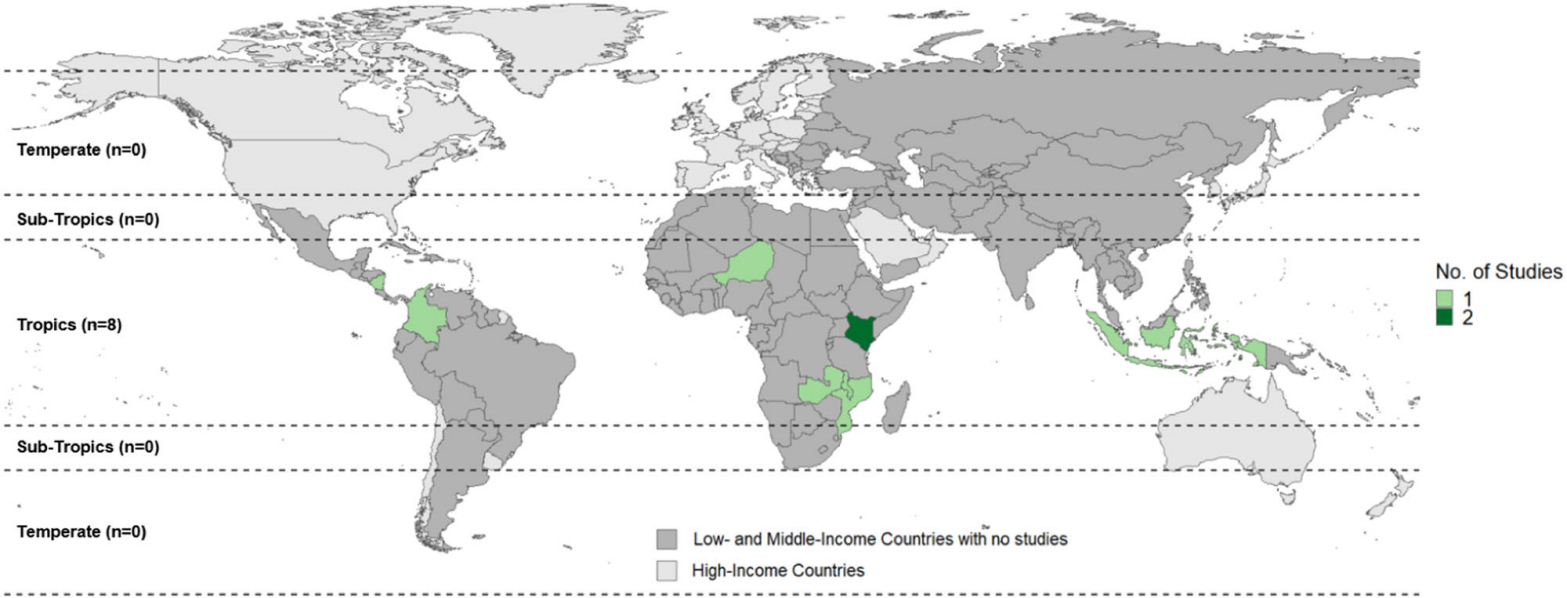
Distribution of intervention impact evaluation studies by country climatic zone

The intervention studies included in this EGM are spread across tropical L&MICs (Figure 4), with Sub‐Saharan Africa having the most countries with a study (*n* = 5, 63%). There were two studies (25%) conducted in Latin America and the Caribbean, and one study (13%) conducted in East Asia and Pacific. Kenya was the only country where more than one study was conducted (*n* = 2, 25%). The countries with impact evaluations included: Colombia, Indonesia, Kenya, Malawi, Mozambique, Nicaragua, and Zambia. All fall within the tropics. In Figure 4, countries in grey are L&MICs where no relevant studies on agroforestry interventions were found.

Five of the eight included impact evaluations (63%) presented results disaggregated by at least one measure of equity (Table 5). Three of the seven studies presented results disaggregated by two or more measures of equity, one of which presented results disaggregated by all four measures captured in this EGM. The most common groups disaggregated in the agroforestry intervention studies were socioeconomic level and educational level (*n* = 4 each) with three studies disaggregating results by gender and one by race/ethnicity.

#### 6.2.2 Overall empirical evidence on agroforestry interventions

The eight studies described above, we identified an additional 32 observational studies of agroforestry interventions. These 32 studies evaluated the impact of an agroforestry intervention against a control group (adopter/nonadopter or before‐after), but the study design used nonrandom assignment (not an experimental design) and did not use a quasiexperimental approach to adjust for nonrandom assignment to estimate a treatment effect.

This section discusses all 40 studies on agroforestry intervention impacts we identified. Most of the studies were published as journal articles (*n* = 32, 80%), with three published as book chapters (8%), two published as organization reports (5%), and one each as a conference proceeding, discussion paper, and thesis. The countries with interventions studied include Kenya (*n* = 8, 20%); India (*n* = 5, 13%); Indonesia and Malawi (*n* = 4, 10% for each); Bangladesh, Brazil, and Indonesia (*n* = 3, 8% for each); Nepal (*n* = 2, 5%); and Bolivia, Colombia, Ethiopia, Ghana, Mexico, Mozambique, Nicaragua, and Zambia (*n* = 1, 2% for each). That Kenya had the most studies was expected since World Agroforestry Center (ICRAF) is headquartered there. The most common world‐region for the intervention studies was Sub‐Saharan Africa (*n* = 16, 40%), followed by South Asia (*n* = 10, 25%), and finally Latin America and the Caribbean (*n* = 7, 18%) and East Asia and Pacific (*n* = 7, 18%).

##### Distribution of studies across interventions

**Figure 5:**
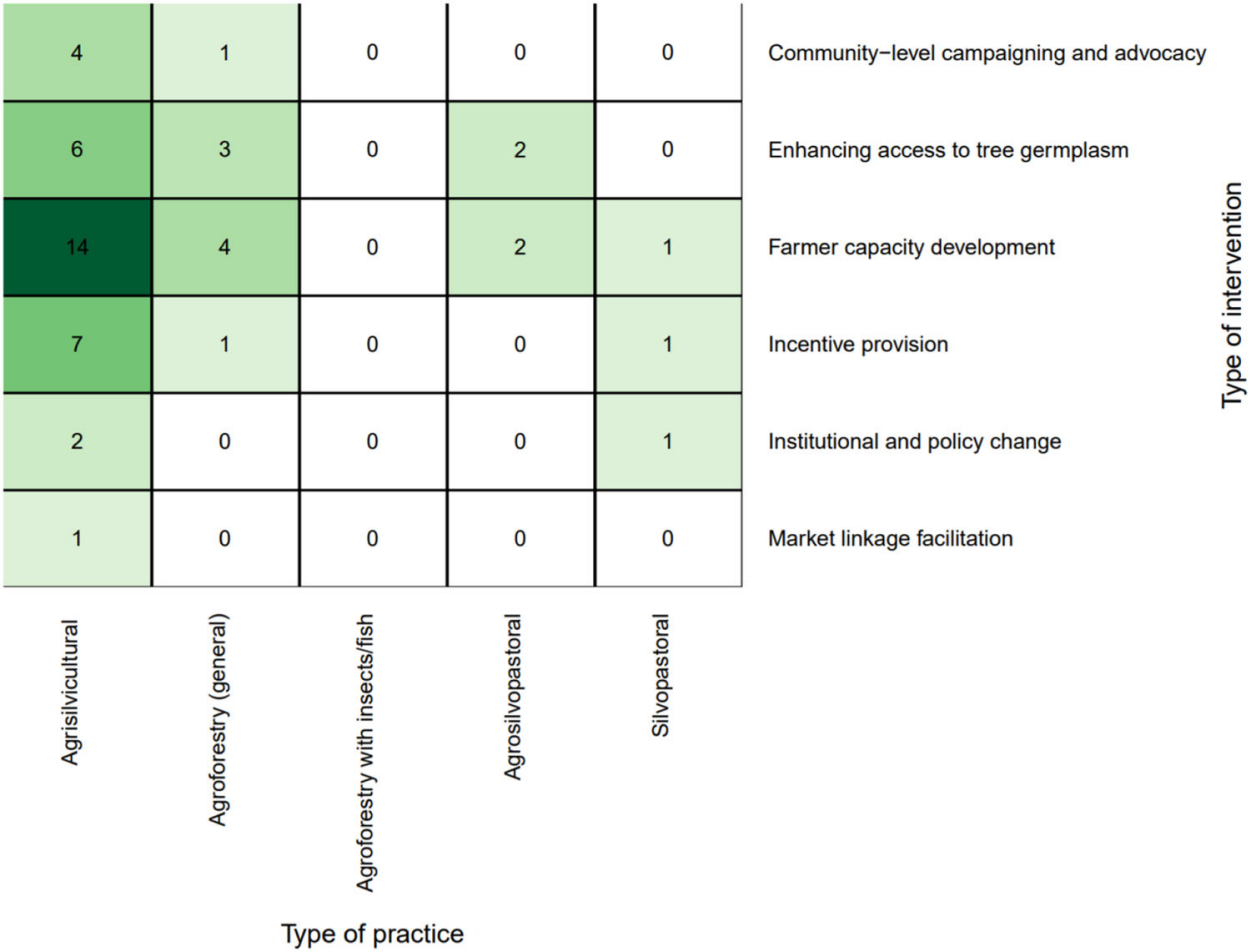
Distribution of interventions and practices

**Figure 6:**
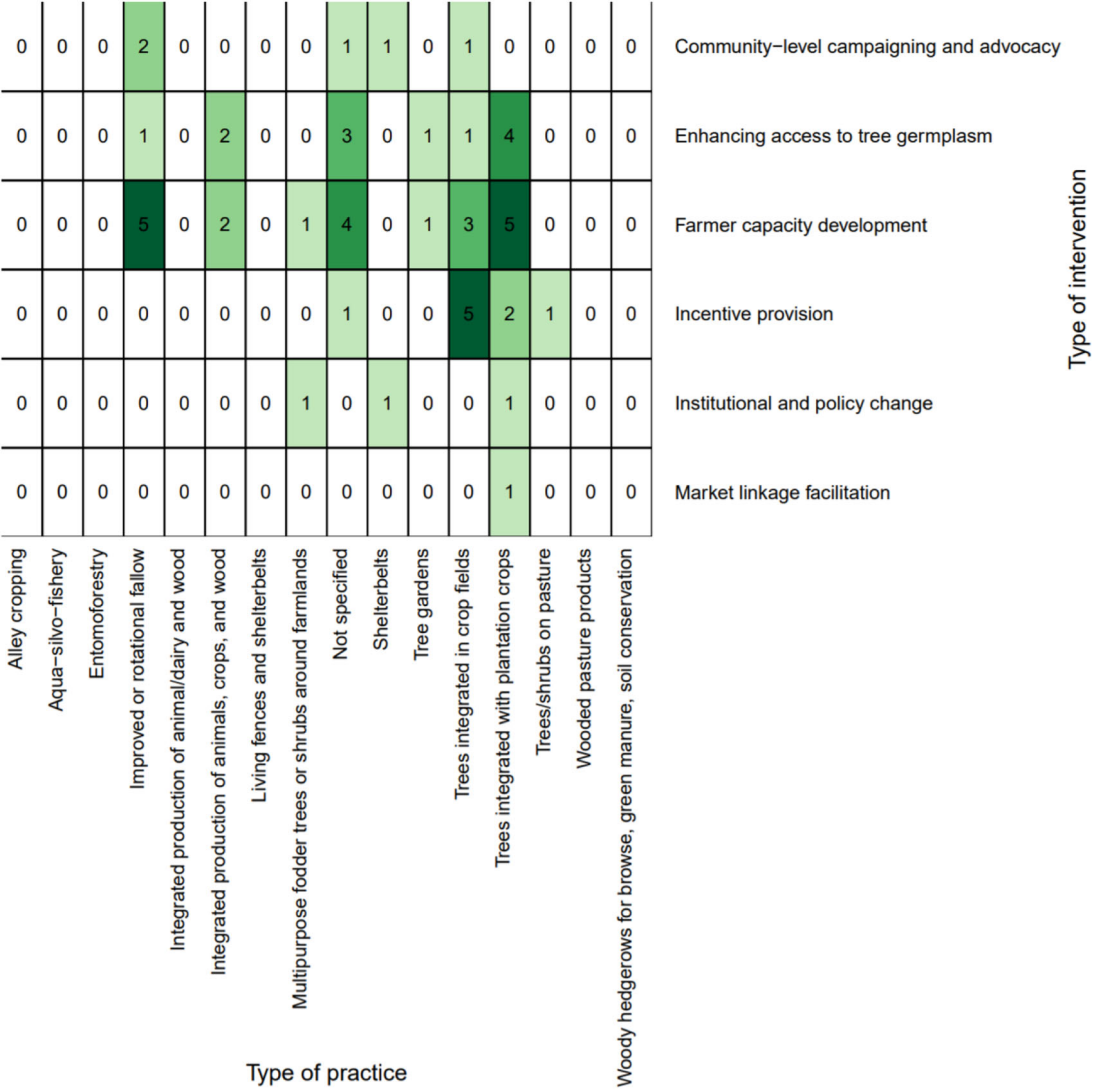
Distribution of interventions and specific practices

*Farmer capacity development* was the most common intervention type (*n* = 21, 53%) (Figure 5). The most studied practices for all interventions were *agrisilvicultural* practices (*n* = 34, 85%) (Figure 5), with the most common types of specific practices being *improved or rotational fallow* and *trees integrated with plantation crops* (*n* = 5, 13% for each) (Figure 6). *Incentive provision* interventions to promote *trees integrated in crop fields* (*n* = 5, 13%) was the other most common intervention‐specific practice linkage.

##### Distribution of studies across outcomes assessed

**Figure 7:**
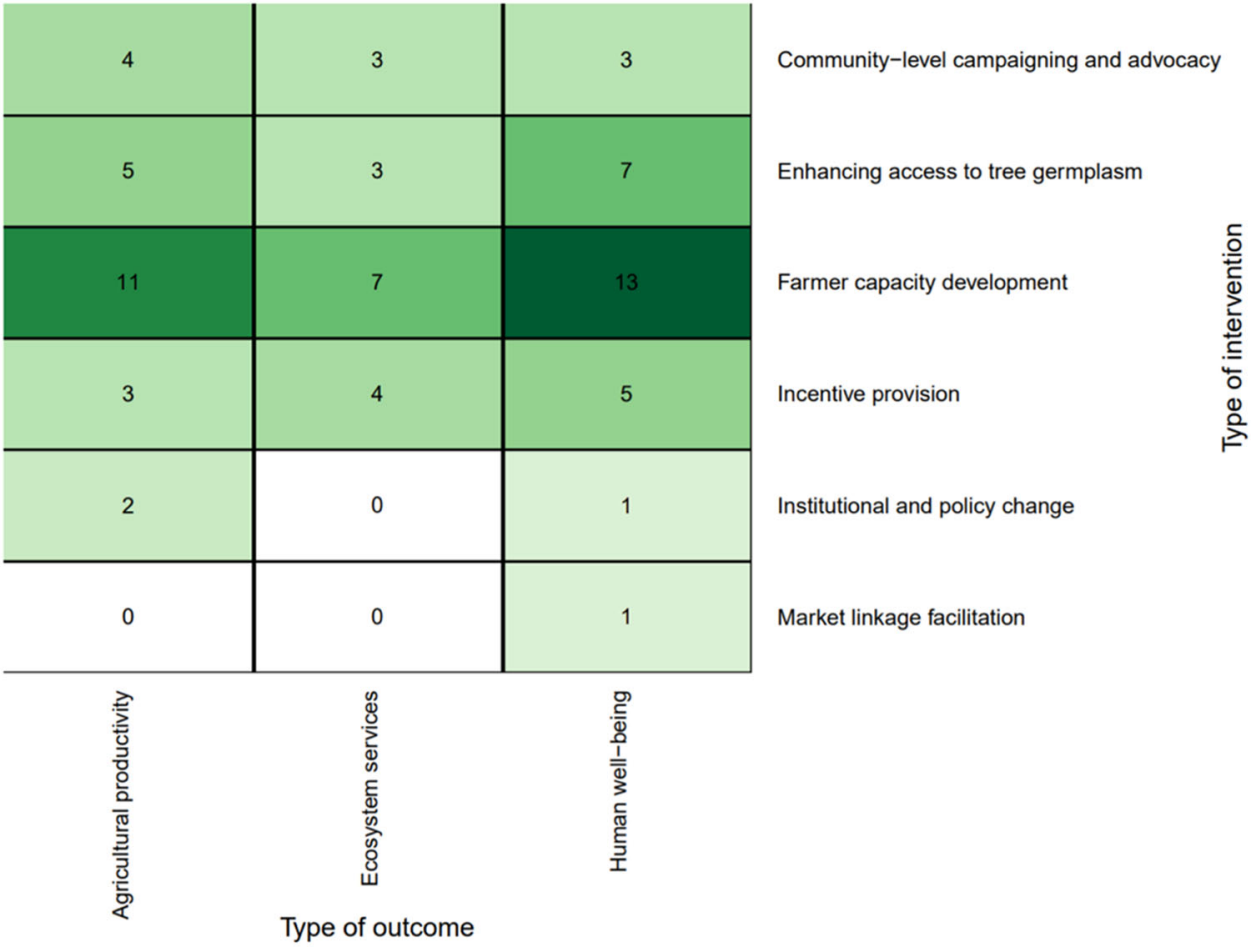
Distribution of interventions and outcomes

**Figure 8:**
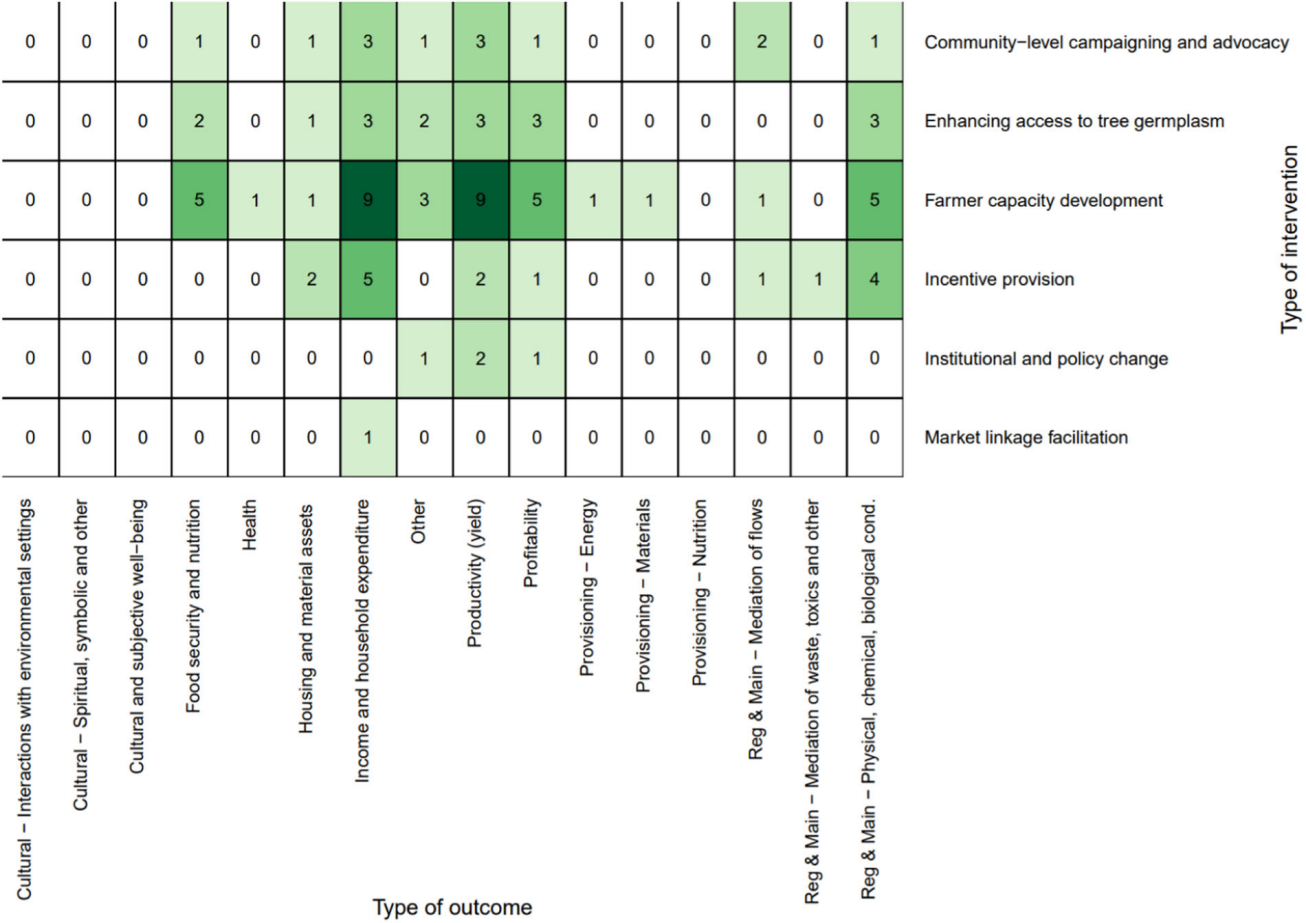
Distribution of specific outcomes and interventions

The most studied linkages for the intervention studies were *farmer capacity development* with *human well‐being* (*n* = 13, 33%), followed by *farmer capacity development* with *agricultural productivity* (*n* = 11, 28%) (Figure 7). Regarding the specific outcomes studied, Figure 8 shows that the most‐studied linkages were *farmer capacity development* with *productivity (yield)* and *income and household expenditure*.

### 6.3 Characteristics and trends of studies on outcomes of agroforestry practices

This section presents the characteristics of empirical studies assessing of the adoption of agroforestry *practices* without evaluating an intervention designed to promote such adoption. These studies compare agroforestry practices against conventional agricultural or forestry practices for at least one of the outcome categories considered in this EGM. We identified 344 such practice studies (out of the 384 total empirical studies).

Nearly all of the practice studies used a correlational study design, comparing a group of farmers adopting a practice with a group of farmers that did not (*n* = 342). These studies used multivariate regression analysis or other quantitative or qualitative methods comparing adoption against a control without any attempt to adjust for nonrandom assignment between treatment and control groups. The remaining two studies did attempt to control for potential confounders, with one using PSM (Haglund, Ndjeunga, Snook, & Pasternak, 2011) and the other using a randomized complete block design in an one on‐farm trial (McDonald, Healey, & Stevens, 2002). The latter study examined treatment plots managed by different farmers in Jamaica to compare the relative impacts of four land uses: secondary forest, bare soil, agriculture, and agroforestry intercropping crops with trees in contour hedges.

#### 6.3.1 Distribution of studies across practices

**Figure 9:**
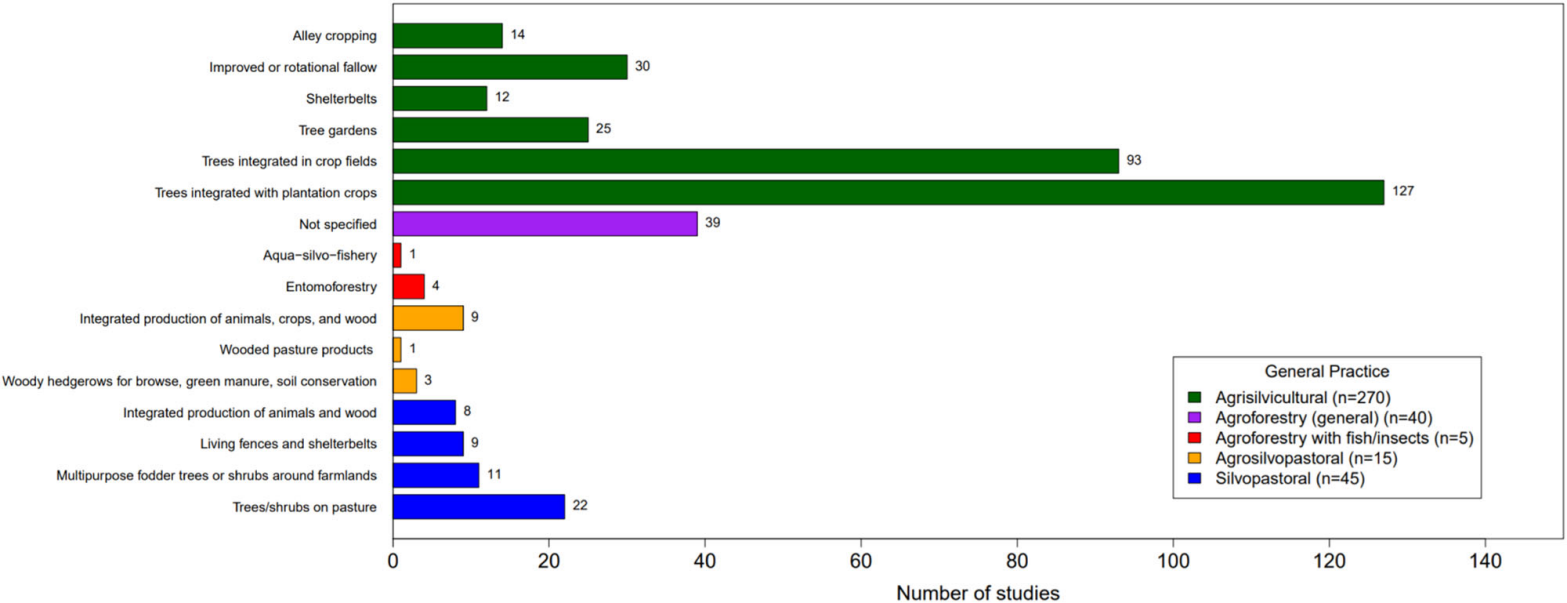
Distribution of studies by agroforestry practice

Figure 9 shows the distribution of studies by agroforestry practice described. The most common general practice type was agrisilvicultural (78%; 270 studies), followed by silvopastoral agroforestry (13%, 45 studies), and general agroforestry (unspecified type) (12%, 40 studies). Agrosilvopastoral practices were assessed in 15 studies (4%), with only one study focusing on agroforestry with fish/insects (<1%).

When looking at more specific practices (Figure 9), trees integrated with plantation crops was the most common (37%, 127 studies), followed by trees integrated with crop fields (27%, 93 studies). Both comprise part of the broader agrisilvicultural category. In the silvopastoral category, the most common practice was trees and shrubs in pastures (6%, 22 studies). The two least frequently studied practices in our review were aqua‐silvo‐fishery and wooded pasture products, with one study each.

#### 6.3.2 Distribution of studies across outcomes assessed

**Figure 10:**
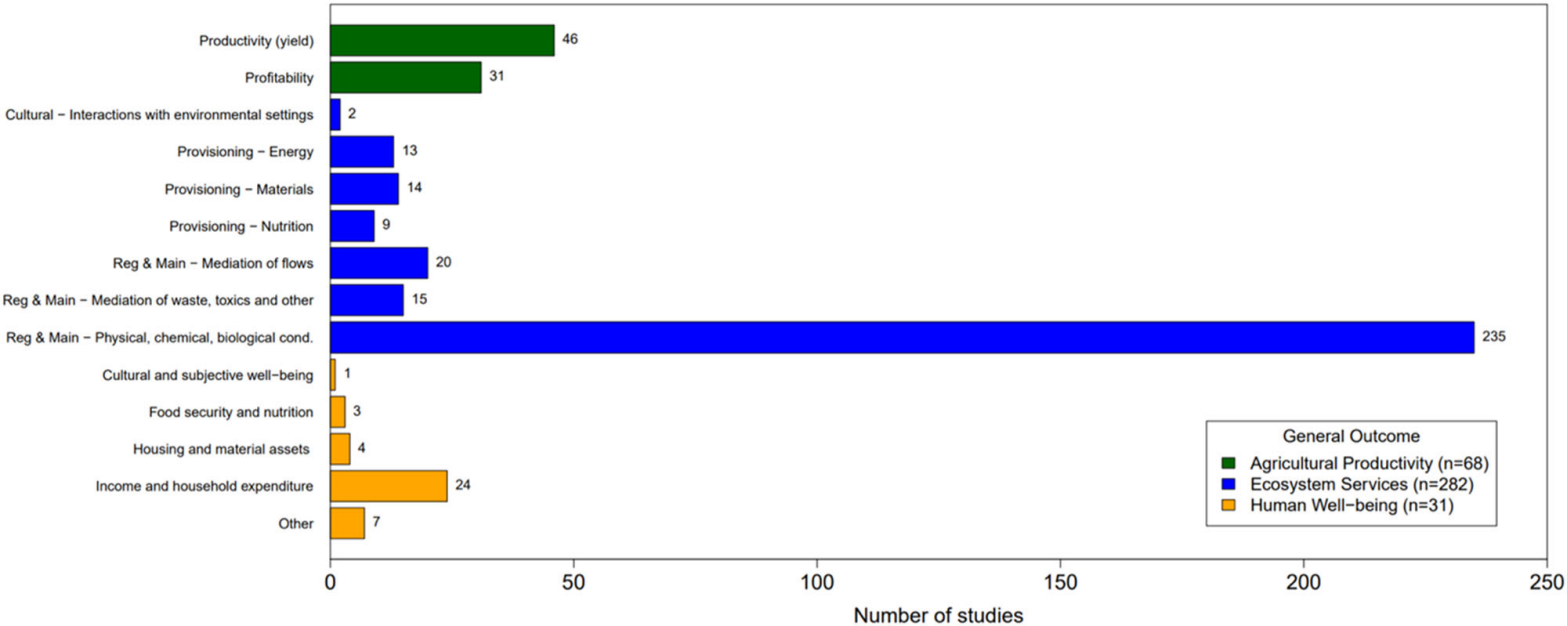
Distribution of studies by outcome

Figure 10 shows the distribution of studies by agroforestry outcomes assessed. Ecosystem services was by far the most commonly assessed general outcome category (*n* = 282, 82%) followed by agricultural productivity (*n* = 68, 20%) and human well‐being (*n* = 31, 9%). The most commonly studied specific outcome was the regulation and maintenance of physical, chemical, and biological conditions (*n* = 235, 68%; Figure 10). The second most common specific outcome was agricultural productivity yield (*n* = 46, 13%). The third most common specific outcome was household and income expenditure (*n* = 24, 7%).

**Figure 11:**
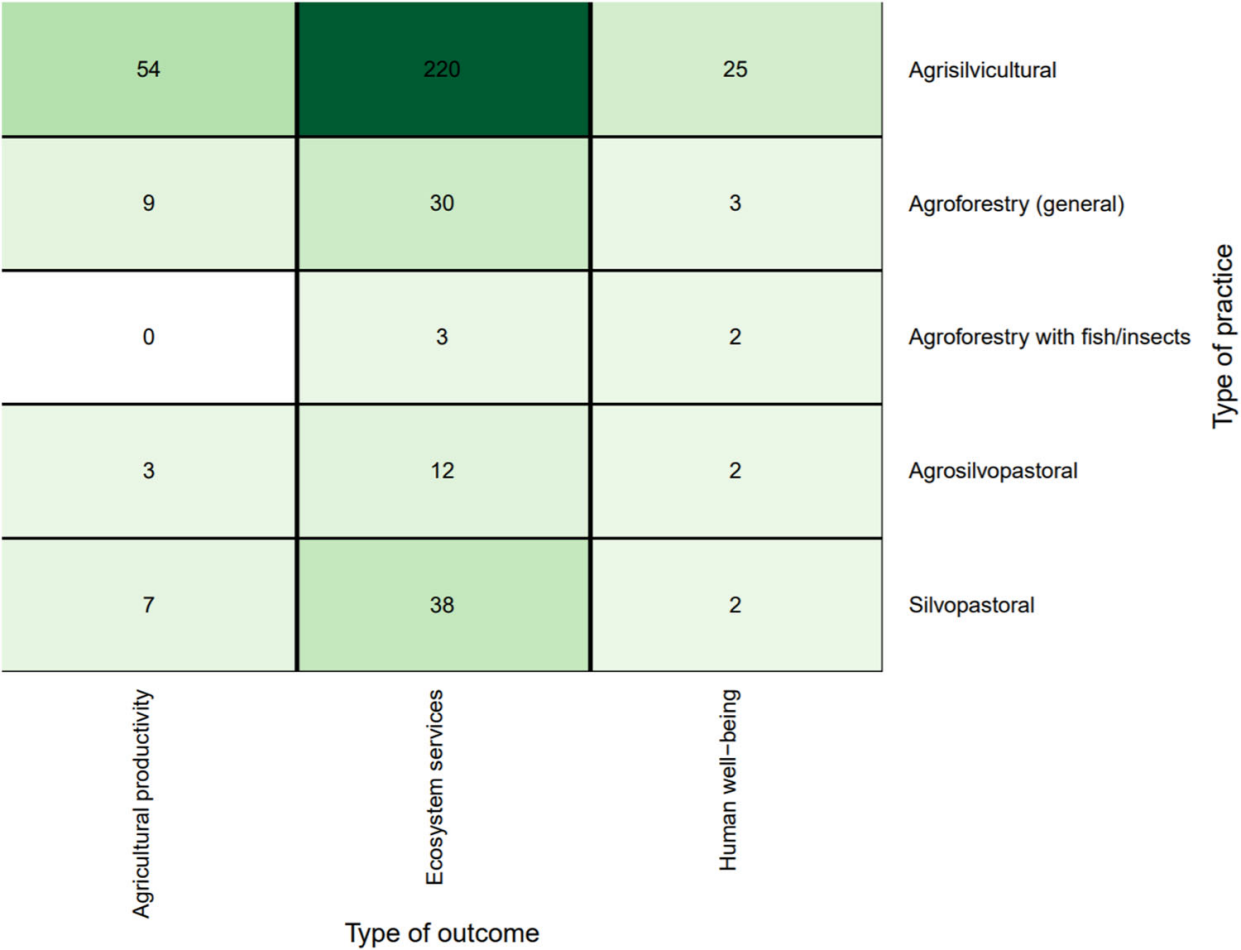
Distribution of studies by practices and outcomes

Looking at the combination of practices and outcomes (Figure 11) shows that the majority of studies that focus on agrisilvicultural practices examined ecosystem services outcomes (*n* = 220, 64%). The second most common outcome for agrisilvicultural practices was agricultural productivity (*n* = 54, 16%).

**Figure 12:**
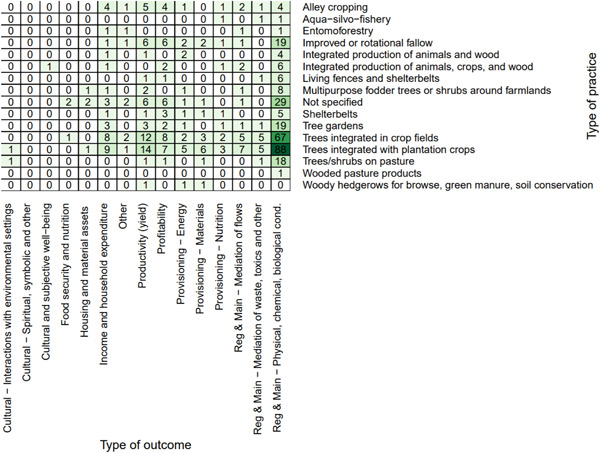
Distribution of studies by specific practices and outcomes

Figure 12 shows the diversity of more specific linkages between practices and outcomes. The most studied linkage was the correlation of trees integrated with plantation crops on the regulation and maintenance of physical, chemical, and biological conditions (*n* = 91, 24%). The second most common practice for studies that focused on this outcome were trees integrated in crop fields (*n* = 71, 19%). A further 31 studies did not provide more specific information on the agricultural practice associated with this ecosystem service‐related outcome.

This heat map reveals a concentration of studies assessing the correlation of practices that integrated trees with plantation crops or integrated trees in crop fields on agricultural yield and on income and household expenditure. At the same time, it shows some major gaps, with many linkages poorly explored or not examined at all. In particular, there appears to be very little evidence on the nonincome‐related dimensions of human well‐being, such as health, nutrition, and cultural and subjective well‐being. Among ecosystem services outcomes studied, our map reveals a focus on regulating and maintenance rather than provisioning.

#### 6.3.3 Geographic distribution of practices studies

**Figure 13:**
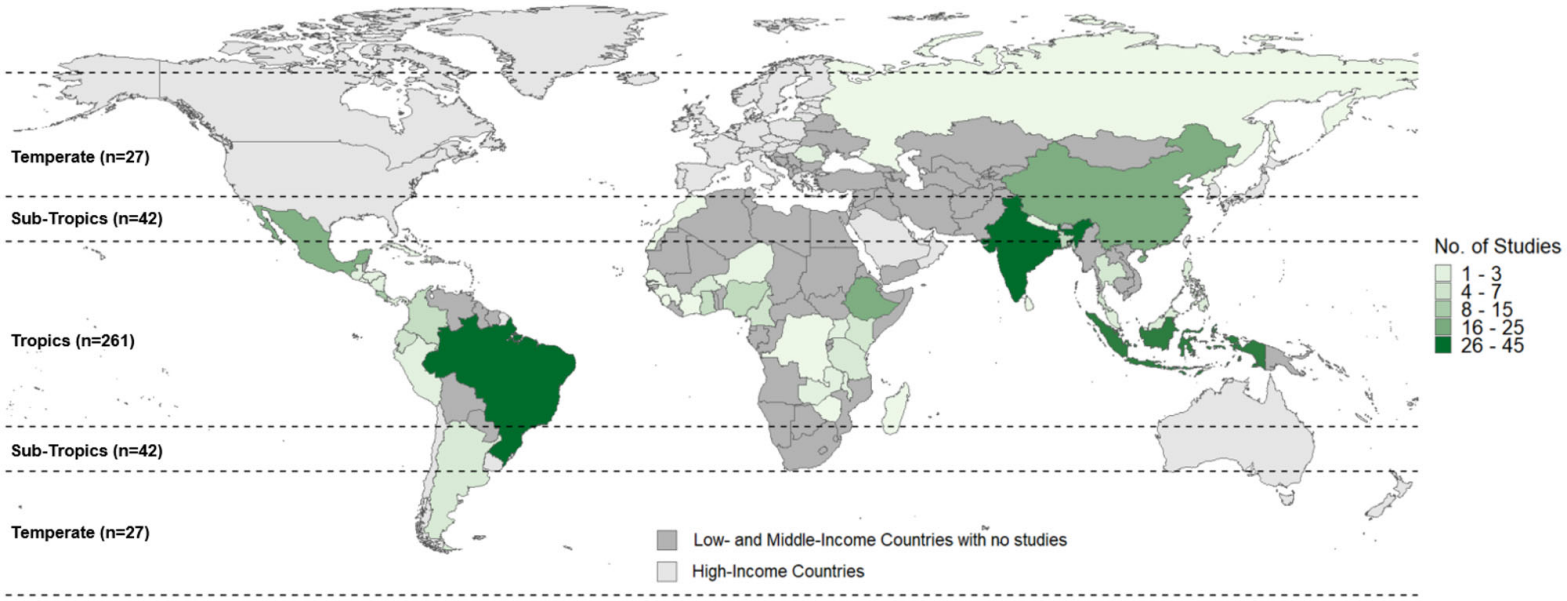
Distribution of practice studies by country and climatic zone

**Figure 14:**
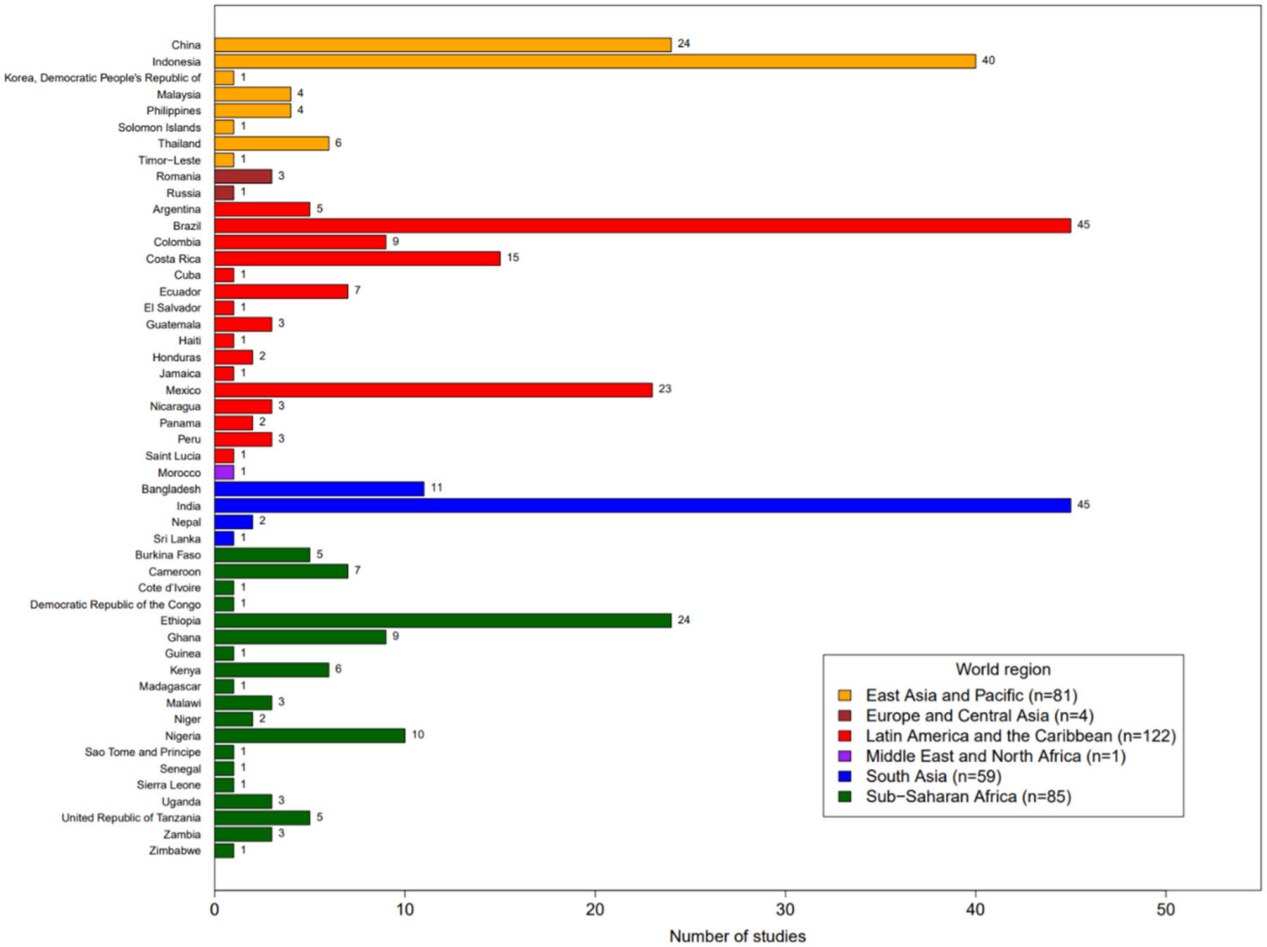
Distribution of practice studies by country

This EGM includes 344 practice studies from across L&MICs in different world regions (Figures 13 and 14). Latin America and Caribbean was the most studied region with 122 practice studies (35%). The second most studied region in this EGM was Sub‐Saharan Africa with 85 studies (25%), followed by East Asia and Pacific with 81 practice studies (24%) and South Asia with 59 practice studies (17%). Europe and Central Asia and Middle East and North Africa had four and one practice studies respectively (<1% for both).

Within the regions, countries were unevenly represented. In South Asia, for instance, 94% of the practice studies in the region were in India (*n* = 45) and Bangladesh (*n* = 11), while Nepal (*n* = 2) had the remaining part of the practice studies. The region of East Asia and Pacific shows similar results. Indonesia (*n* = 40) and China (*n* = 24) comprised 77% of the practice studies from that region. In Latin America and Caribbean, 66% of the studies were in Brazil (*n* = 48), Mexico (*n* = 23), and Costa Rica (*n* = 15), and in Sub‐Saharan Africa 67% of the practice studies were in Ethiopia (*n* = 24), Nigeria (*n* = 10), Ghana (*n* = 9), Cameroon (*n* = 7), and Kenya (*n* = 6).

Studies of agroforestry practices were from three major climatic zones (Figure 13). The tropics were the most represented zone with 261 practice studies (76%). Twelve percent of the practice studies were in subtropical regions (*n* = 42) and 8% were from temperate regions (*n* = 27). Four percent of the practice studies were from countries that included multiple major climatic zones (*n* = 15).

#### 6.3.4 Distribution of studies by literature type and publication date

**Figure 15:**
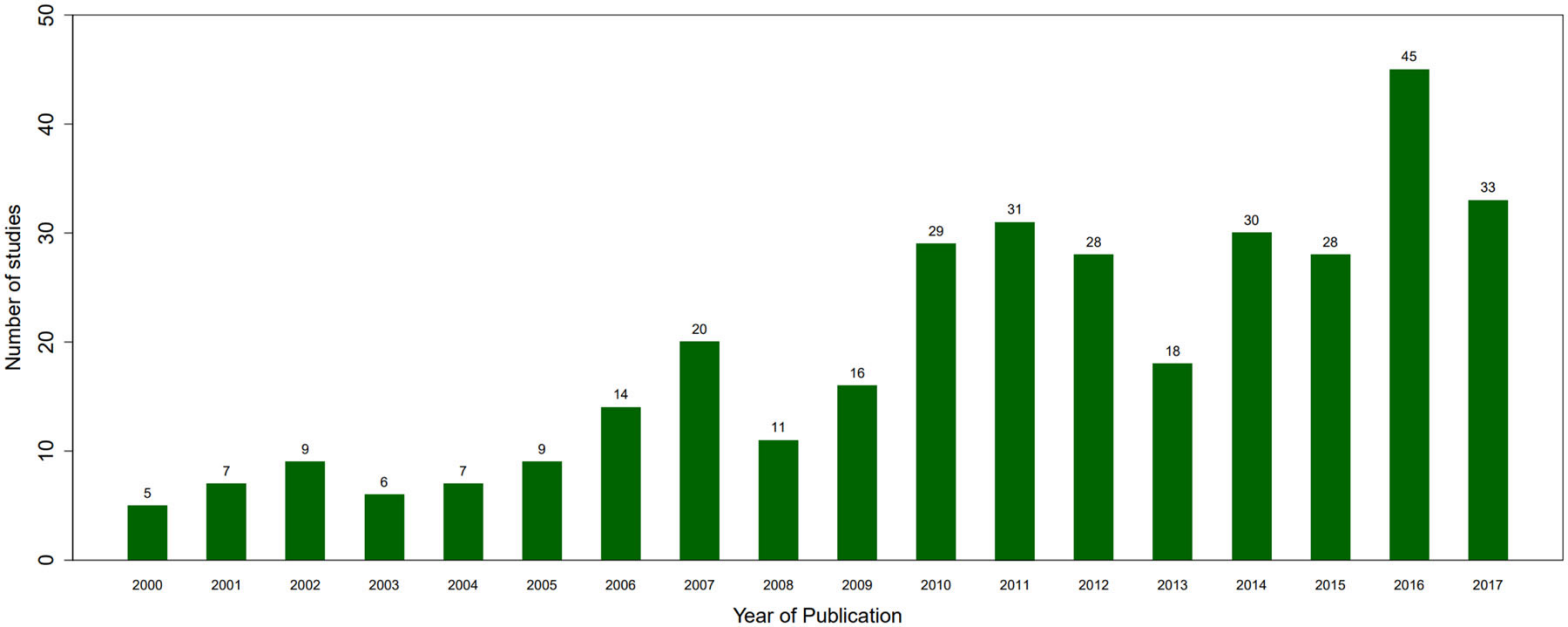
Empirical evidence for practices by publication date. The year 2017 only includes studies through June 30th

Practice studies included in this EGM were conducted from the year of 2000 to mid‐2017. More practice studies were conducted in the last 8 years (68%), than in the first decade (32%). In 2000, only five practice studies were conducted (1%), but that number increased to 31 practice studies in 2011 (9%) and reached a peak in 2016 with practice 45 studies (13%). We note that this upward trend likely continued in 2017 as data for 2017 only extend to the first six months of the year (Figure 15).

There were three publication types for practice studies included in this EGM. The clear majority of the practice studies included are journal articles, with 331 practice studies (96%). Out of the 14 remaining practice studies, 10 are book chapters (3%) and four are conference proceedings (1%).

#### 6.3.5 Distribution of studies by subpopulation

Only 11 (3%) of the practice studies included results that were disaggregated by different subpopulations. Of these, socioeconomic level and gender were the most studied, with seven and six studies, respectively, followed by literacy/education level (*n* = 4). Only one study examined results by race/ethnicity.

### 6.4 Characteristics and trends of the evidence base from SRs

Twelve SRs that fit the inclusion criteria were identified. None of the identified reviews included evidence relating to interventions. Table 6 provides detailed information on each of the 12 SRs included.

**Table 6:** Description of systematic reviews

Title	Publication year	Countries	Regions	Review design	Confidence	Number of impact evaluations	Practices	Outcomes	References
Cereal yield response to conservation agriculture practices in drylands of West Africa: A quantitative synthesis	2012	Burkina Faso, Mali, Niger, Senegal	Sub‐Saharan Africa	Meta‐analysis	Low	63 studies	*Agrisilvicultural* Trees integrated in crop fields	Productivity (yield)	Bayala et al. (2012)
Carbon sequestration and net emissions of CH_4_ and N_2_O under agroforestry: Synthesizing available data and suggestions for future studies	2016	Kenya, Panama, Indonesia, Philippines, Togo, India, Canada, Senegal, Tanzania, China, Malawi, Costa Rica, France, Mali, USA, Puerto Rico, Zambia, Germany, Cameroon, Mexico, Chile, UK, Zimbabwe, Brazil, Peru	Worldwide	Meta‐analysis	Low	71 publications (135 data sets)	*Agrisilvicultural* Silvopastoral Trees integrated in crop fields Trees integrated with plantation crops Tree gardens Shelterbelts Alley cropping Trees/shrubs on pasture Living fences and shelterbelts Improved or rotational fallow	Reg & Main—Mediation of waste, toxics and other nuisances	Kim et al. (2016)
Biodiversity function and resilience in tropical agroforestry systems including shifting cultivation	2016	Tropics	Tropics	Vote‐counting	Low	146 articles	*Agrisilvicultural* Agroforestry (general) Trees integrated with plantation crops Tree gardens Improved or rotational fallow Not specified	Reg & Main—Physical, chemical, biological conditions	Norgrove and Beck (2016)
Effects of agroforestry on pest, disease and weed control: A meta‐analysis	2015	Colombia, Kenya, Ghana, Mexico, Cameroon, Nigeria, Indonesia, Vietnam, Uganda, Philippines, Tanzania, UK, Ethiopia, Costa Rica, Panama, Western Samoa, Zambia, USA, Ecuador	Worldwide	Meta‐analysis	Low	42 studies	*Agrisilvicultural* Agroforestry (general) Trees integrated in crop fields Trees integrated with plantation crops Tree gardens Alley cropping Improved or rotational fallow Not specified	Reg & Main—Physical, chemical, biological conditions	Pumariño et al. (2015)
A meta‐analysis reveals mostly neutral influence of scattered trees on pasture yield along with some contrasted effects depending on functional groups and rainfall conditions	2013	Kenya, Senegal, USA, Australia, Portugal, Spain, Tanzania, South Africa, Chile	Worldwide	Meta‐analysis	Low	27 studies	*Silvopastoral* Trees/shrubs on pasture	Provisioning—Nutrition	Rivest et al. (2013)
Shaded coffee and cocoa—double dividend for biodiversity and small‐scale farmers	2017	Mexico, Guatemala, El Salvador, Nicaragua, Ghana, Cameroon, Indonesia, Costa Rica, Ecuador, Peru, Panama, Brazil, Rwanda, Tanzania	Tropics	Meta‐analysis	Low	23 studies (10 cases)	*Agrisilvicultural* Trees integrated with plantation crops	Productivity (yield) Profitability Reg & Main—Physical, chemical, biological conditions	Jezeer et al. (2017)
Meta‐analysis of maize yield response to woody and herbaceous legumes in Sub‐Saharan Africa	2017	Kenya, Tanzania, Malawi, Zimbabwe, Zambia, Ghana, Benin, Ethipia, Nigeria, Rwanda, Uganda, Togo, Cameroon, Burkina Faso	Sub‐Saharan Africa	Meta‐analysis	Low	94 peer‐reviewed publications	*Agrisilvicultural* Trees integrated in crop fields Improved or rotational fallow	Productivity (yield)	Sileshi et al. (2008)
A global meta‐analysis of the biodiversity and ecosystem service benefits of coffee and cacao agroforestry	2013	Ecuador, Indonesia, Mexico, Colombia, Cameroon, Costa Rica, Brazil, Ethiopia, Guatemala, Jamaica, India, El Salvador, Panama, Ghana, Dominican Republic	Africa, Latin America and Asia	Meta‐analysis	Low	74 studies	*Agrisilvicultural* Trees integrated with plantation crops	Reg & Main—Physical, chemical, biological conditions General ecosystem services (unspecified)	De Beenhouwer et al. (2013)
Trees for life: The ecosystem service contribution of trees to food production and livelihoods in the tropics	2017	India, Kenya, Brazil, Indonesia, Sudan, Nigeria, Malawi, Ghana, Cote d'Ivoire, Costa Rica, Cameroon, Uganda, Tanzania, Mexico, Australia, Zambia, Sri Lanka, Malaysia, Malawi, Ethiopia, China, Burkina Faso, Benin, Argentina	Tropics	Systematic review	Low	74 peer‐reviewed publications	*Agroforestry (general)* Not specified	Productivity (yield)	Reed et al. (2017)
The effect of afforestation on water infiltration in the tropics: A systematic review and meta‐analysis	2007	Cameroon, Nigeria	Sub‐Saharan Africa	Meta‐analysis	Low	2 studies (6 experiments)	*Agroforestry (general)* Not specified	Reg & Main—Mediation of waste, toxics and other nuisances	Ilstedt et al. (2007)
The 4 per 1000 goal and soil carbon storage under agroforestry and conservation agriculture systems in Sub‐Saharan Africa	2018	Burkina Faso, Ethiopia, Kenya, Malawi, Nigeria, Senegal, Sudan, Swaziland, Tanzania, Uganda, Zambia, Zimbabwe	Sub‐Saharan Africa	Meta‐analysis	Low	15 studies	*Agrisilvicultural* Trees integrated in crop fields Alley cropping Improved or rotational fallow	Reg & Main—Physical, chemical, biological conditions	Corbeels et al. (2018)
The impact of swidden decline on livelihoods and ecosystem services in Southeast Asia: A review of the evidence from 1990 to 2015	2017	Laos, Thailand, Vietnam, Malaysia, Indonesia, Philippines, Myanmar, Papua New Guinea	Southeast Asia	Systematic review	Medium	93 studies	*Agrisilvicultural* Improved or rotational fallow	Reg & Main—Physical, chemical, biological conditions Productivity (yield) Income and household expenditure Cultural and subjective well‐being	Dressler et al. (2017)

Based on this appraisal, 11 of the SRs included in this EGM were reviews rated low confidence, and one was rated as medium confidence. The primary concern was the risk of bias arising through the methodology, reporting, and lack of risk of bias analysis of included studies within the reviews. All 12 reviews all used a defined, systematic search, but the searches were typically not comprehensive given, for example, a limited set of search terms used, limited databases consulted, and lack of consideration of grey literature. They also rarely incorporated risk of bias or heterogeneity analyses. Finally, the methods used for combining data were also not clearly explained in many of the reviews.

**Figure 16:**
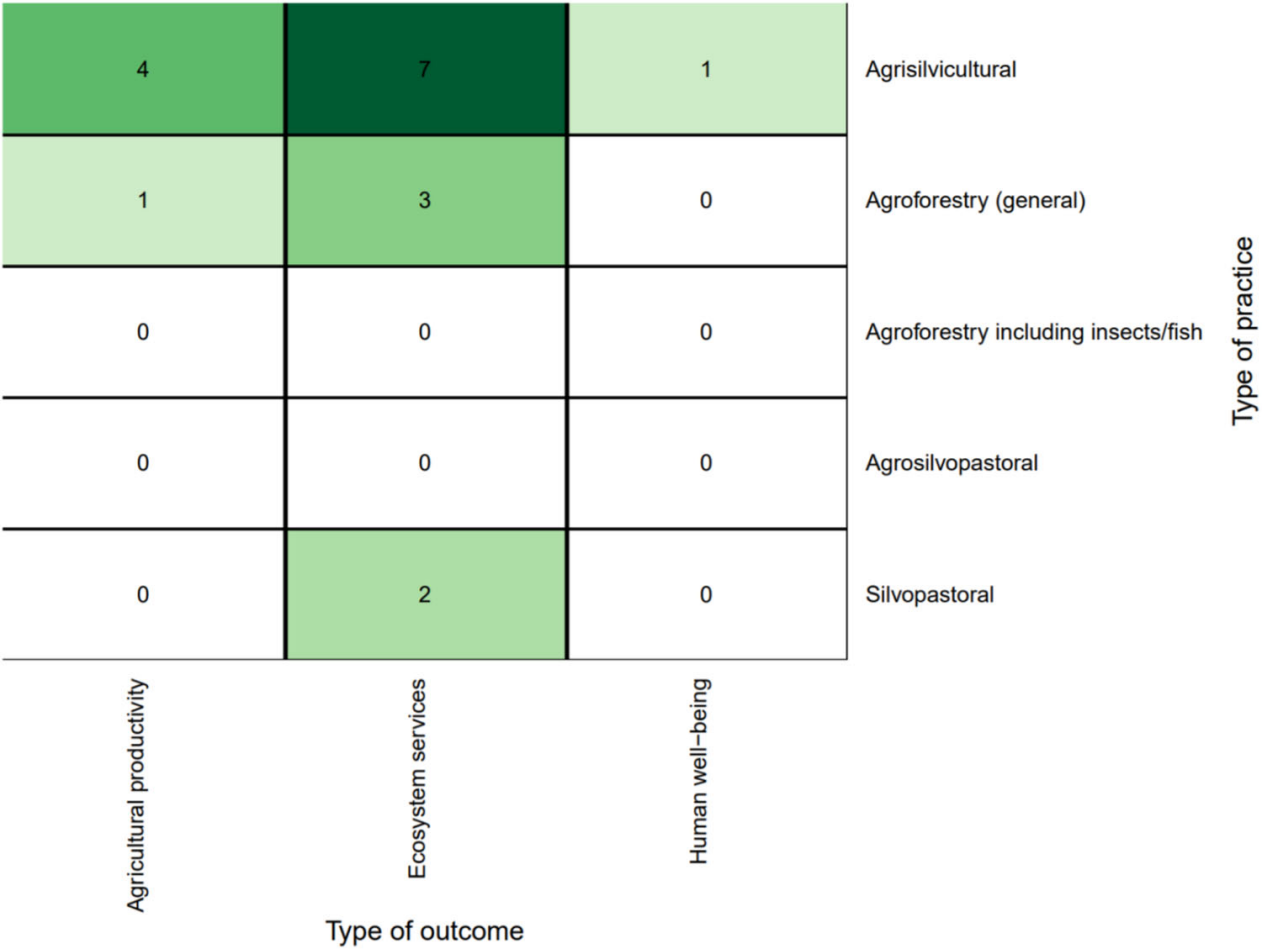
Distribution of systematic reviews by practices and outcomes

Most of the 12 included reviews discussed multiple practices, with only a few looking at multiple outcomes. Figure 16 summarizes all data found by number of instances in each practice category and outcome type. Table 6 shows the breakdown of the specific practice and outcome types. If one review stated a practice with two different outcome types or a single outcome with multiple practices it would be counted multiple times in Figure 16. Across the SRs there were a total of 33 different combinations of practice and outcome types. Improved or rotation fallow was the most common agroforestry practice occurring in 50% of studies (*n* = 6) examined, followed by trees integrated with plantation crops and trees integrated in crop fields (*n* = 5 each, 42% each). Regulation and Maintenance—Physical, Chemical, and Biological Conditions was the most common outcome type, representing 52% of studies (*n* = 6).

Most of the reviews were meta‐analyses, as shown in Table 6. While meta‐analysis occurred 75% of the time, there were also two SRs and one vote‐counting. All of the included studies used a systematic search strategy. We note an additional 39 review‐type studies were identified, but these 39 reviews did not use systematic search strategies.

### 6.5 Concentration of evidence and gaps

The main EGMs highlighted above for interventions (Figure 3) and practices (Figure 12) highlight a number of important gaps.

#### 6.5.1 What are the major evidence gaps?

##### 6.5.1.1 An “absolute gap” in the evidence on the impacts of agroforestry interventions

The most notable evidence gap relating to the evidence mapped in this EGM is the lack of evidence on the effects of agroforestry interventions. We identified eight quasiexperimental studies and no experimental studies evaluating the effects of agroforestry interventions. Looking at the main EGM (Figure 3) and more detailed map (Figure 8) highlights a large number of empty or near empty cells. While a few intervention‐outcome intersections include a handful of studies there is no area that can be described as saturated. Similarly, given the limited evidence base overall, there are major and widespread gaps in the geographic coverage of intervention studies. Existing studies focus on measuring human well‐being outcomes, with fewer studies assessing effects on agricultural productivity and ecosystem services.

The limited evidence base was not entirely unexpected. Our decision to also map studies of practices in the absence of an intervention was partially due to our expectation that there would be few impact evaluations available. But the low number of studies in this category was still surprising. In particular, we were surprised to find that no studies on Costa Rica's nationwide payment for ecosystem services program, which includes an agroforestry component (Porras, Barton, Cascante, & Miranda, 2013), were identified. Additionally, the lack of intervention studies is especially notable given the large volume of literature on specific agroforestry practices and widespread promotion of and investment in agroforestry. It is also somewhat surprising given that intervention studies are now widespread in the sister fields of agriculture and forestry. For example, a recent EGM on agricultural innovation (Lopez‐Avila, Husain, Bhatia, Nath, & Vinaygyam, 2017) included more than 300 completed impact evaluations while the EGM by Puri et al. (2016) on forest conservation interventions identified 110 impact evaluations. In line with our findings, however, the latter study found only two impact evaluations on agroforestry, both of which are in our EGM.

We believe there are several reasons for these gaps. First, the location of agroforestry at the intersection agriculture and forestry has often meant that the research communities of each field neglect agroforestry, focusing instead on concerns more core to the respective field. Second, agroforestry has often taken place through autonomous adoption based on traditional practices in many L&MICs rather than being promoted explicitly through government policies. Government interest is changing, but seeing agriculture and forestry separately has been the historic norm, as indicated by the structure of government itself, with agencies responsible for these two domains often separated. Finally, there is often a significant lag between the adoption of agroforestry practices or systems, and measurable outcomes. Therefore, a complete evaluation requires a long‐term commitment that increases the cost of such studies.

The lack of evidence on agroforestry interventions underscores the need for more high‐quality impact evaluation studies that use experimental or quasiexperimental designs. Agroforestry has been promoted and supported by many agencies worldwide, yet there exists little evidence on the effect of this support on desired outcomes such as agricultural productivity, ecosystem services, and human wellbeing. Impact evaluations of agroforestry policies, programs, and projects implemented on farmers land are urgently needed to help us understand what types of interventions work, under what circumstances, and with what effects for different objectives and social groups. Such studies should assess effects on the range of relevant outcomes along the causal chain, including agricultural productivity, ecosystem, and human well‐being outcomes.

##### 6.5.1.2 A relatively large literature on adoption of agroforestry practices

In contrast to the paucity of evidence on the effects of agroforestry interventions there is a relatively large literature of studies assessing the relationship between adoption of agroforestry practices and relevant outcomes, agricultural productivity in particular. The practice with the least amount of evidence is that of agroforestry including fish (aqua‐silvo‐fishery), where only one study met our inclusion criteria. We expect that this agroforestry practice is not especially prevalent on farmers’ land, which explains why it has not been much studied. The second least researched category of practices is agrosilvopastoral, which we expect may be more prevalent in the world and may be more deserving of further investigation.

The agroforestry practice studies were concentrated in India, Brazil, and Indonesia followed by China, Mexico, and Ethiopia. Together, these six countries were the focus for 55% of the studies on agroforestry practices. Regionally, Africa has a relatively large number of studies, but if we exclude the two countries with the most studies (Ethiopia and Nigeria), the continent would be well below the other regions. Half of the 14 countries in Africa where studies took place only had one study. More generally, there were hardly any studies in the L&MICs of Europe and Central Asia, and Middle East and North Africa.

Finally, we also note a lack of equity focus in the literature, both for intervention and practice studies. Given the focus on ecosystem service and productivity outcomes for the practice studies, this is an expected finding. The intervention studies, however, focus more on human well‐being outcomes, and while we find that five of the eight impact evaluation studies include some measure of equity in their analysis in the form of subgroup analysis, there is a lack of more substantive equity analysis. Future impact evaluation studies should incorporate consideration of equity in their design and analyses of effects on human well‐being outcomes.

##### 6.5.1.3 Synthesis gaps: No high‐quality SRs

All included SRs (*n* = 12) addressed practices rather than interventions. This result mirrors what we have found in conducting this EGM, with the majority of the included studies relating to practices. However, 11 of the SRs on practices were rated as low confidence. Therefore, any area of evidence concentration presented in our map offer areas with potential for SR. Despite the overall paucity of evidence on agroforestry intervention effectiveness, a potentially useful SR would be one that synthesizes the evidence on the most prevalent and promising practices (considering also including field trials), combined with a synthesis of relevant interventions mechanisms used in agriculture more broadly. Together, this evidence could identify what practices appear most efficacious and how to promote uptake of such practices among farmers.

We suggest the synthesis of the evidence on practices should focus on the broad range of relevant agricultural productivity and ecosystem services outcomes, perhaps leaving out human wellbeing outcomes. This is because much of the evidence will be based on correlational studies, and human wellbeing outcomes in particular may suffer from selection bias (wealthier and more educated farmers more likely to adopt new practices). It would also be important that any synthesis of this evidence consider key variables likely to drive heterogenous outcomes, including type of climate, agricultural system, and crop type. Additionally, the evidence from agroforestry field trials should be synthesized similarly in conjunction with or separately from on‐farm research.

A SR of the currently available quasiexperimental impact evaluation studies would also be worthwhile. Carrying out such syntheses would provide baseline insights to inform future policy and programming relating to agroforestry interventions and also present an important baseline for future research. This kind of synthetic work is also needed to help address what seems to be a persistent dichotomy in agroforestry research between studies in ecology and agronomy, which tend to focus on the agricultural productivity and environmental outcomes of agroforestry practices, and studies in international development that emphasize human well‐being outcomes of agroforestry interventions.

## 7 DISCUSSION

### 7.1 Summary of main results

Agroforestry has been widely practiced, promoted, and studied across the L&MICs of Africa, Asia, and Latin America. Given its prevalence and promise, agroforestry is promoted for its potential to provide a vital contribution to advancing several of the 2030 UN SDGs (Van Noordwijk et al., 2018; Waldron et al., 2017). Indeed, high‐level policy documents in many L&MICs now explicitly call for the integration of trees into farming systems (FAO 2013) and international donors have invested billions of dollars in agroforestry interventions around the world (AidData 2017; Tierney et al., 2011).

In this study, we have presented the findings of an EGM that used systematic methods to identify, collect, and visually portray available evidence on the effects of agroforestry in L&MICs on three important outcomes: agricultural productivity, ecosystem services, and human well‐being. Our EGM differs from other such maps in that it describes evidence not only on interventions to promote agroforestry but also on specific agroforestry practices, whether they have been promoted through specific programs or not. These different literatures largely correspond to the type of research typically conducted in ecology/agronomy and international development, respectively.

### 7.2 Limitations and potential biases in the review process

A central finding of this review is that the evidence base on the impacts of agroforestry interventions on farmers’ land remains very limited. While we identified 384 studies in total, only eight addressed the effects of interventions, with remaining literature consisting of observational studies of agroforestry practices as compared to nonagroforestry land use. Thus, the evidence backing claims about the potential of for agroforestry to improve agricultural productivity, ecosystem services, and human well‐being is lacking.

### 7.3 Agreements and disagreements with other studies or reviews

Agroforestry has often been overlooked in research and policy on agriculture and rural development (Miller, Ordonez, et al. 2017). The focus of agriculture is usually on annual crops and trees are usually considered the domain of forestry. However, forestry largely concerns itself with trees in forests rather than outside them. Without more reliable evidence on intervention pathways and impacts, agroforestry risks further marginalization, thereby undermining progress on broader development and sustainability goals. Below we draw out implications of this and other findings for research, policy, and practice.

## 8 AUTHORS’ CONCLUSIONS

### 8.1 Implications for practice and policy

Given that the major finding is that there is a near absolute gap in evidence on the effects of the agroforestry interventions, it may seem there are not many implications for policy and practice. In a sense this is true—we lack systematic understanding of the relative effectiveness of different interventions to inform new policies and programs. However, the overall findings of this report do suggest some important paths forward.

From a donor perspective, the EGM highlights major areas where there is a need to support more primary research, particularly on specific kinds of agroforestry interventions, as well as where evidence synthesis might be conducted. These two areas are detailed in earlier sections. Relatedly, there is a major opportunity for donors and governments and other partners to work together to support and implement randomized controlled trials (RCTs) of different agroforestry interventions to enhance our understanding of what works and what does not seem to work in this area.

### 8.2 Implications for research

The results of our study show that there is a significant need for further research on the socioeconomic and ecological effects of agroforestry. This need relates to synthesis of the existing evidence base on the impacts of both agroforestry interventions and practices as well as new primary research on agroforestry interventions.

Our study reveals that rigorous evidence on the effects of agroforestry interventions remains extremely limited. Impact evaluations of agroforestry interventions remain challenging due to the long time scale between implementation and impacts. Trees take a long time to grow, and the resulting effects on environmental health and human livelihoods may take decades. The scope of many development projects usually only lasts a few years, so long‐term monitoring and evaluation must be built in to project proposals and designs. Many studies we found only examined whether farmers adopted agroforestry as the results of an intervention, without measuring the subsequent impacts on social‐ecological outcomes. One approach to addressing the need for long‐term evaluation is establishing on‐farm experimental trials, for which there may be better justification for long‐term monitoring proposals. Finally, RCTs are rarely conducted in agroforestry research based on our findings, but RCTs can offer valuable insights into how agroforestry interventions impact farmer livelihoods and the environment.

The complexity that comes with integration of agricultural, forest, and pastoral, and other systems, as done in agroforestry, poses significant challenges to evaluating the effectiveness of specific agroforestry interventions. However, given the potential of agroforestry to contribute to a number of major SDGs simultaneously, there is an urgent need for such impact evaluation. Nevertheless, there are examples demonstrating such evaluation is possible. Expanding the number of impact evaluations of agroforestry interventions, especially using RCTs, therefore, represents a major opportunity for expanding and improving the existing evidence base.

A better understanding of the win‐win scenarios and tradeoffs associated with agroforestry is urgently needed, particularly given the potential of agroforestry to help achieve the SDGs. More robust evidence on the different environment and development objectives agroforestry can advance, including climate change mitigation and adaptation, poverty reduction, and health and nutrition, is needed in its own right, but also to enable analysis of synergies and tradeoffs.

Agroforestry encompass a huge suite of different practices that are flexible in their design and composition. This spectrum of practices that agroforestry captures makes it difficult to define for comparison to alternative land uses. Coupling the ecological suitability of different agroforestry practices with the associated impacts on human well‐being, instead of leaving the ecology and human well‐being outcomes separate, could build understanding of the complex dynamics of agroforestry to help design better agroforestry interventions. We need to better understand the costs and benefits of agroforestry from an interdisciplinary perspective, incorporating economics, social science, and environmental science, to assess its viability as a conservation practice while also considering the needs of farmers.

## INFORMATION ABOUT THIS REVIEW

## ROLES AND RESPONSIBILITIES

Content: D. C. M. is an expert on forest and natural resource policy and has conducted research on the socioeconomic contributions of forests and trees in Africa, Asia‐Pacific, and Latin America. K. B. is specialist in evaluating the effectiveness of agriculture, conservation and natural resource policy interventions on global food security, the environment, and social welfare. K. H. leads Monitoring, Evaluation and Impact Assessment the ICRAF and has extensive knowledge of agroforestry policy, practice, and research. P. J. O. has conducted research on the effect of forest and agroecological management practices in Central America, Colombia, Mexico and Sub‐Saharan. S. E. B. has carried out parallel research on agroforestry impacts in high‐income countries and brings more general knowledge on agroforestry practices in different contexts around the world.

EGM methods: D. C. M., K. B., P. J. O., and S. E. B. have substantial understanding of EGM methods. The team consulted with Birte Snilstveit, and leading expert in EGM methods, throughout the EGM process.

- Statistical analysis: D. C. M., K. B., P. J. O., and S. E. B. have expertise in econometrics and statistics. S. E. B. created the R code for the analysis and figures.
- Information retrieval: P. J. O. and S. E. B. led the information retrieval and data management. N. J. N. and S. F. helped with data collection. The team consulted an advisory group of experts in agroforestry and systematic searches to help create the search string. The team also consulted library scientists at the University of Illinois at Urbana‐Champaign for guidance in searching multiple academic databases and grey literature sources.
- Report: D. C. M., P. J. O., S. E. B., S. F., N. J. N., K. H., and K. B. wrote the report.

## Supporting information

Supplementary information

Supplementary information

## Footnotes

1 We note that the total percentage here, and at different points throughout this report, can sum to more than 100% as a given study could include more than one intervention and outcome.

## APPENDIX A SEARCH STRATEGY AND DATABASES

The databases that we searched for publications were:

- SCOPUS
- EBSCO: Econlit
- OVID: Agricola
- Web of Science: Core Collection
- Web of Science: CAB Abstracts and Global Health
- AGRIS

The search terms used in each database can be found in Table A1 (constructed using the terms from CAB thesaurus and also the EGM framework described above). Each search string included each of the agroforestry practices from Table 1. These terms and search strings were modified through a scoping exercise in Web of Science, SCOPUS, and EBSCO, where the search terms were used and the results were evaluated against a set of 40 relevant studies assembled by the team. We note that the intervention types are more generic, including topics well beyond agroforestry, so our search focused on practices.

**Table A1:** Search terms by intervention and outcomes

Category	Terms
Practices	(“agroforest*” OR “agriforest*” OR “agro‐forest*” OR agrosilvicultur* OR agrisilvicultur* OR “improved fallow*” OR “shade tree*” OR “rotational tree fallow*” OR parkland* OR “multipurpose tree*” OR “tree garden*” OR “forest garden” OR “alley cropping” OR intercropping OR “shifting cultivation” OR shelterbelt* OR “natural vegetation strip*” OR “wind break*” OR “sloping agricultural land technology” OR “hedgerows” OR “hedge cropping” OR silvopastoral* OR silvipastoral* OR “fodder tree*” OR “living fence*” OR “integrated animal and wood production” OR “trees on pasture” OR agrosilvopastoral* OR “integrated production of animals, crops and wood” OR “tree‐crop‐livestock” OR “apiculture with trees” OR entomoforestry OR “aqua‐silvo‐fisher*” OR “multi‐purpose tree lot*” OR “tree* on farms” OR “orchard” OR “on‐farm tree*” OR “woody hedgerows” OR “wooded pastures produce” OR “fertili*er trees” OR “shade species” OR “shade‐grown” OR “alternative agriculture” OR “tree‐based system*” OR “tree fallow*” OR “planted fallow*” OR woodlot* OR “boundary planting” OR “mixed trees and crops” OR “conservation agriculture with trees” OR “farmer managed natural regeneration” OR homegarden OR “fodder shrub*” OR “multi‐strata systems” OR “nitrogen fixing trees”)
Study designs	AND (“impact” OR “outcome” OR “result” OR “effect*” OR “intervention” OR “evaluation” OR “assessment” OR “*effectiveness” OR “cost‐benefit” OR “cost benefit” OR “efficacy” OR “systematic review” OR “field trial” OR “observational stud*” OR “trial” OR “random* control* trial*” OR “random* trial*” OR RCT OR “propensity score matching” OR PSM OR “regression discontinuity design” OR RDD OR “difference in difference*” OR matching OR (random* adj3 allocat*) OR “instrumental variable*” OR IV OR evaluation OR assessment OR “comparison group” OR counterfactual OR “counter factual” OR counter‐factual OR quasi‐experimental OR quasiexperimental OR ((quantitative or experiment*) adj3 (design OR study OR analysis)))

Additionally, in order to identify the existing grey literature, the websites of various organizations that are likely to produce published and unpublished research were searched, using the search terms from Table A1. The list of relevant research organizations (Table A2) has been constructed from cross‐validation of websites listed in the systematic mapping protocols of agroforestry related studies (e.g., Bottrill et al., 2014; Leisher et al., 2016; Nguyen, Herbohn, & Clendenning, 2015). This list was validated with the external EGM advisory group. To optimize the scope of the search while ensuring transparency in our methods, we followed the approach developed by Haddaway et al. (2017), which allowed us to search multiple websites simultaneously and to extract the relevant information from each website into a single database. Finally, we also contacted key informants within 3ie, ICRAF, and other relevant organizations for identification of additional relevant literature for screening and inclusion.

**Table A2:** List of websites from relevant organizations

Organizations	Websites
Australian Centre for International Agricultural Research (ACIAR)	http://aciar.gov.au/aboutus
Center for International Forestry Research (CIFOR)	http://www.cifor.org
Economy and Environment Program for Southeast Asia (EEPSEA)	www.eepsea.org
Food and Agriculture Organization (FAO)	http://www.fao.org
French Agricultural Research Centre for International Development	www.cirad.fr
GFIS	www.GFIS.net
IDEAS RePEc (Research Papers in Economics)	https://ideas.repec.org
Inter‐American Development Bank (IADB)	www.iadb.org
International Food Policy Research Institute Library (IFPRI)	http://library.ifpri.info/
International Institute for Environment and Development	http://www.iied.org
International Impact Initiative (3ie)	http://www.3ieimpact.org/
International Tropical Timber Organization	www.itto.int
Overseas Development Institute	https://www.odi.org/
Tropical Agricultural Research and Higher Education Center (CATIE)	http://www.catie.ac.cr/en/
United Nations Development Programme (UNDP)	http://www.undp.org
United States Agency for International Development (USAID)	http://www.usaid.gov
USAID Development Experience Clearinghouse	dec.usaid.gov
World Agroforestry Center (ICRAF)	www.worldagroforestry.org
World Resources Institute	http://www.wri.org/

## APPENDIX B DATA EXTRACTION FORMS

**Table B1:** Data extraction form

Variable codes	Variables	Instructions
0.0	No.	Unique ID for each article
0.1	ID	Unique ID for each article (corresponding to eppi)
0.2	Reviewer	Name of reviewer conducting data extraction
0.3	Date of Assessment	Date of data extraction
**1**	**Bibliographic Information**	
1.1	Reference information	Harvard style bibliographic reference
1.2	Publication type	For example, journal article, book chapter, conference paper, thesis, organization report
1.3	Author(s)	Author information in the following format: Last name, first initial.
1.4	Year of publication	Included years 2000 through 2017
1.5	Title	Title of the study/title of book chapter
1.6	Journal/Book title	Name of the journal/book where it was published
1.7	Volume	Journal volume
1.8	Affiliation of first author	Name of university, organization, working group, or other where first author is affiliated at time of publication
1.9	Country of first author affiliation	Location by country of first author affiliation
1.10	Funding	Sources of funding listed for the study
**2**	**Study design and basic information**	***Answer the following questions for each type of intervention or practice in the study***
2.1	Study description	Short description of study
2.2	Multicomponent intervention	Does the intervention have multiple components? (Y/N)
2.3	Intervention type(s)	CHOOSE FROM DROPDOWN—MULTIPLE SELECTION ALLOWED List the intervention(s) used to promote agroforestry (None = The study does not evaluate an intervention)
2.4	Multiple practices	Yes or No
2.5	Practice type(s) (general)	CHOOSE FROM DROPDOWN—MULTIPLE SELECTION ALLOWED List the agroforestry practice(s) studied
2.6	Practice type(s) (specific)	CHOOSE FROM DROPDOWN—MULTIPLE SELECTION ALLOWED List the specific agroforestry practice(s) studied
2.7	Study country/ies	Country(ies) in which the study takes place (where practice/intervention is implemented)
2.8	Study location(s)	Specific location(s) within countries where the study focused. Record smallest spatial scale reported (e.g., community, subnational political unit, regions within country, etc.)
2.9	Type of ecoregion	Tropical, subtropical, and temperate Choose multiple when study spans multiple ecoregions
2.10	Study design type and rating	5 levels: Level 1 (correlation), level 2 (before and after with no comparable control condition), level 3 (b and a with comparable control condition), level 4 (b and a, multiple experimental units, comparable control condition, with control variables for the outcome), level 5 (random assignment and control conditions).*
2.11	Quasiexperimental methods used (if appropriate)	For levels 2 ‐4 studies, type of quasiexperimental method used: difference‐in‐difference, propensity score matching, instrumental variables, regression discontinuity design, panel data regression methods
2.12	Sample size	Number in sample (number of studies, for systematic reviews SRs)
2.13	Sample unit	For example, individuals, households, groups (relevant studies, for SRs)
2.14	Other outcome influences	Potential effect modifiers or inequality (including gender) reported that may have influenced the outcome
2.15	Comparator	Comparator is agriculture, primary (native, natural) forest, secondary forest/plantation, or multiple
2.16	Comparator type	For example, randomized BACI, nonrandomized BACI, temporal, spatial, other (none = correlation studies)
2.17	Disaggregation based on gender?	Yes or No
2.18	Disaggregation based on socioeconomic level?	Yes or No Is the population disaggregated based on socioeconomic level? (assets, income, expenditure, land holdings, or other asset or income based measure)
2.19	Disaggregation based on race/ethnicity?	Yes or No Is the population disaggregated based on race/ethnicity?
2.20	Disaggregation based on literacy/educational level?	Yes or No Is the population disaggregated based on literacy/education level?
**3**	**Outcomes**	***Answer the following questions for each type of outcome in the study***
3.1	Type of outcome	Ecosystem services, Human well‐being, Agricultural productivity
3.2	Subtype of outcomes	CHOOSE FROM DROPDOWN Can choose several of these, for each type of outcome
3.3	Group examined for outcome	Indicate which group is being examined for this outcome (if study is disaggregated by a group)
3.4	Indicator variables used to measure outcomes	For each type of outcome, what are the variables used to measure it
3.5	Outcome data type	CHOOSE FROM DROPDOWN Qualitative, Quantitative, or Both as data type for the outcome
3.6	Direction of the outcome	CHOOSE FROM DROPDOWN Positive or negative (for each subtype of outcome)
3.7	Outcome direction notes	Additional details about the reported outcome direction
**4**	**Mechanisms** ****INTERVENTIONS ONLY****	****ONLY COMPLETE MECHANISMS SECTION IF INTERVENTION, NOT FOR PRACTICE ONLY STUDIES**
4.1	Are there any mechanisms in the study stated as linking the intervention to the outcome?	Yes or No
4.2	Stated mechanism	Describe the mechanism, if stated in the study
4.3	Stated type of model	Theory of change, results chain, logic model, conceptual framework
4.4	How is the model employed	Framing the study, choosing indicators, validating model with data, using data to infer model

Abbreviation: BACI, before‐after control‐impact.
